# Bias-free estimation of information content in temporally sparse neuronal activity

**DOI:** 10.1371/journal.pcbi.1009832

**Published:** 2022-02-11

**Authors:** Liron Sheintuch, Alon Rubin, Yaniv Ziv

**Affiliations:** Department of Neurobiology, Weizmann Institute of Science, Rehovot, Israel; University Medical Center Hamburg-Eppendorf Center for Molecular Neurobiology Hamburg: Universitatsklinikum Hamburg-Eppendorf Zentrum fur Molekulare Neurobiologie Hamburg, GERMANY

## Abstract

Applying information theoretic measures to neuronal activity data enables the quantification of neuronal encoding quality. However, when the sample size is limited, a naïve estimation of the information content typically contains a systematic overestimation (upward bias), which may lead to misinterpretation of coding characteristics. This bias is exacerbated in Ca^2+^ imaging because of the temporal sparsity of elevated Ca^2+^ signals. Here, we introduce methods to correct for the bias in the naïve estimation of information content from limited sample sizes and temporally sparse neuronal activity. We demonstrate the higher accuracy of our methods over previous ones, when applied to Ca^2+^ imaging data recorded from the mouse hippocampus and primary visual cortex, as well as to simulated data with matching tuning properties and firing statistics. Our bias-correction methods allowed an accurate estimation of the information place cells carry about the animal’s position (*spatial information*) and uncovered the spatial resolution of hippocampal coding. Furthermore, using our methods, we found that cells with higher peak firing rates carry higher spatial information per spike and exposed differences between distinct hippocampal subfields in the long-term evolution of the spatial code. These results could be masked by the bias when applying the commonly used naïve calculation of information content. Thus, a bias-free estimation of information content can uncover otherwise overlooked properties of the neural code.

## Author summary

Neuroscientists interested in understanding the nature of the neural code often apply methods derived from the mathematical framework of information theory to quantify the statistical relationship between neuronal activity and a certain variable of interest. For instance, when studying the neural basis for spatial navigation, it is useful to estimate how much information hippocampal neurons carry about the position of an animal within a specific environment. However, the standard measures for estimating information content suffer from an upward bias when applied to small sample sizes, which may lead to misinterpretation of the data. This bias is more pronounced in data from calcium imaging–a widely used technique for recording neuronal activity–because the activity extracted from the measured calcium signal is sparse in time. In this work, we introduce new methods to correct the bias in the naïve estimation of information content from limited sample sizes and such temporally sparse neuronal activity. We show that our bias-correction methods allow an accurate estimation of the information content carried by the activity obtained from calcium imaging data in both hippocampal and cortical neurons, and help uncover differences in the way information content changes during learning across neural circuits.

## Introduction

A fundamental problem in neuroscience is to understand the nature of the neural code–namely, how much and which type of information is carried by the neuronal activity. Recent advances in Ca^2+^ imaging techniques allow the chronic readout of activity from hundreds of simultaneously recorded neurons in freely behaving mice [1–3] and tracking, with little ambiguity, the same neurons over multiple days [4]. Hence, estimating information content from Ca^2+^ imaging data enables one to investigate the neural code in a population of cells and to study, for example, how the neuronal coding properties evolve over time during the learning of a specific behavioral task. One widely used information-theory-derived approach is to treat neurons as communication channels and quantify the mutual information (MI) between neuronal activity patterns and a specific variable of interest [5–9]. Calculating MI relies on the ability to accurately estimate the conditional probability distributions of firing rates given each stimulus, which requires the collection of very large amounts of data. Due to the finite amount of trials or stimulus repetitions that can be recorded in an experiment, estimations of MI in neuroscientific studies generally suffer from a significant positive error (upward bias) [10–13].

A simple, widely used measure that was developed by Skaggs et al., (*Skaggs information index*) allows one to estimate the amount of information conveyed by the activity of a neuron about a given experimentally measured variable [14,15]. The Skaggs information index (*SI*) is mathematically related to MI, as it is proportional to the first-order approximation of the MI rate at the limit as the time bin approaches zero, under the assumption of Poisson firing statistics [16,17]. When expressed in bit/spike, SI quantifies the tuning specificity of the neuron, and when in bit/sec, it quantifies the rate at which information about the variable of interest is conveyed by the neuron’s activity. In the case that the variable is the animal’s position, the output of the SI quantifies the spatial information carried by the neuronal activity, which has become a standard measure in many studies of neuronal representations of space [18–26]. When the number of samples is limited, SI is a better suited measure than MI, because it relies solely on calculating the neuron’s average firing rate for each value of the stimulus (i.e., *tuning curve*), instead of the full conditional probability distribution of firing rates given each stimulus [27]. Thus, unlike MI, SI does not depend on how the range of observed firing rates is discretized or on the specific choice of temporal binning. This independence becomes crucial when estimating information content from Ca^2+^ imaging data, where the bin size is limited by the recording frame rate, which precludes the estimation of the MI rate at the infinitesimal time bin limit.

Nonetheless, similarly to MI, SI exhibits a significant upward bias due to inaccuracies in the estimation of the tuning curves when sample sizes are limited [15,19,22,28]. Furthermore, Ca^2+^ imaging techniques are less sensitive to the detection of spikes, which may result in temporally sparser neuronal activity compared to electrophysiological techniques [3,29], thereby exacerbating the bias when applying information theoretic measures [15,17]. Numerous methods have been developed to correct the bias in the naïve estimation of MI [11,12,30–34]. However, while these methods allow an unbiased estimation of MI in the asymptotic sampling regime, where sample sizes are sufficiently large [10], most of them do not generalize well to temporally sparse neuronal activity (as that obtained from contemporary Ca^2+^ imaging techniques) or to small sample sizes. Furthermore, no bias-correction method has been adapted or tested on SI, and measurements of SI are typically reported in their naïve, biased form [18–25]. This could lead to data misinterpretation and false conclusions about the quality of the neural code, for instance, when comparing data sets with different magnitudes of bias within a given study or across different studies.

Here, we built upon principles developed to estimate MI in electrophysiology data [10,11,30] to establish two independent methods for correcting the bias in the naïve estimation of information due to temporally sparse neuronal activity and a limited sample size. Focusing on SI which is widely used in studies of spatial cognition, we show that our methods outperform their counterparts in terms of generating an unbiased estimation of information content. Our methods also allow an accurate, unbiased estimation of MI, demonstrating their general applicability to various information measures. We validated the accuracy of the methods using simulated data with known ground-truth information content and Ca^2+^ imaging data recorded from both the mouse hippocampus and primary visual cortex.

Applying our bias-correction methods to data recorded from the hippocampus of freely behaving mice allowed us to estimate the true spatial information content and spatial resolution of hippocampal place cells irrespective of the specific choice of spatial binning. We also revealed that neurons with higher spike rates tend to carry higher average spatial information per spike, and exposed differences in the long-term evolution of the spatial code between hippocampal subfields CA1 and CA3. Importantly, in cases of an insufficient sample size, these results would have been masked by the bias in the naïve calculation of information content, emphasizing the necessity of our methods. We provide a freely accessible GitHub repository with the software for applying our methods for the unbiased estimation of information content using both SI and MI (*https*:*//github*.*com/zivlab/unbiased_information_estimation*).

## Results

### The naïve estimation of information content is positively biased by limited sample sizes and temporally sparse neuronal activity

**Fig 1:**
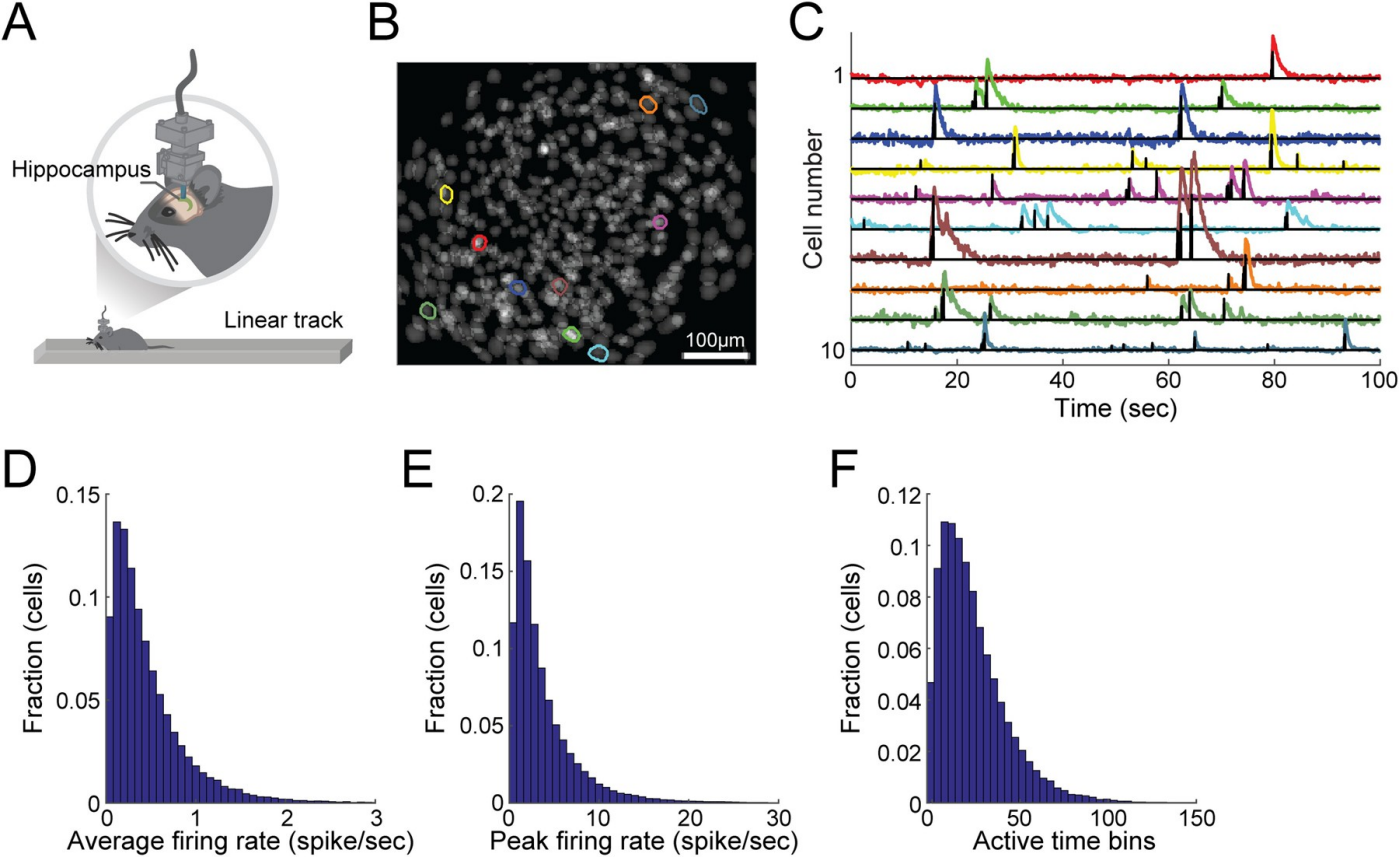
Ca^2+^ imaging from hippocampal neurons during linear-track exploration yields temporally sparse neuronal activity. (A) Ca^2+^ imaging in the hippocampus of freely behaving mice as they run back and forth on a linear track. (B) Ten example contours of detected cells overlaid on the projection of all the detected cells in a given imaging session. (C) The fluorescence traces (colors) extracted for the example cells shown in B and their estimated spike trains (black). (D-F) The distribution of the average firing rates across all positions (D), of the firing rates in the most active position (E), and of the total number of active time bins in a given session (F) for hippocampal place cells. Distributions in D-F are for 44,981 place cells during running periods, pooled from N = 9 mice (1,717–9,906 cells per mouse) recorded over 8 sessions in each of two different environments per mouse. We focused only on sufficiently active cells (≥ 5 active time bins in a given session). Cells were analyzed independently, without tracking them across sessions.

We performed one-photon Ca^2+^ imaging (20 frames/sec) of the hippocampal CA1 and CA3 of freely behaving mice (N = 9) as they repeatedly traversed linear tracks (35.9 ± 10.9 [mean ± SD] track traversals per session in each of the two running directions) across 16 20-minute-long sessions (8 sessions in each of two environments; Fig 1A). We then extracted estimated neuronal spike trains of individual neurons from the imaging data using previously established routines (Fig 1B and 1C) [35]. We focused our analysis on the activity of neurons that were modulated by mouse position (*place cells*) during the running periods [1,36]. The extracted estimated spike trains were temporally sparse, with place cells showing activity in < 1% of the running time bins (average estimated firing rate = 0.47 ± 0.44 spike/sec [mean ± SD], peak estimated firing rate = 3.92 ± 4.30 spike/sec [mean ± SD], and active time bins per session = 26.6 ± 19.1 [mean ± SD] for a temporal bin size of 50 msec; Fig 1D and 1F).

**Fig 2:**
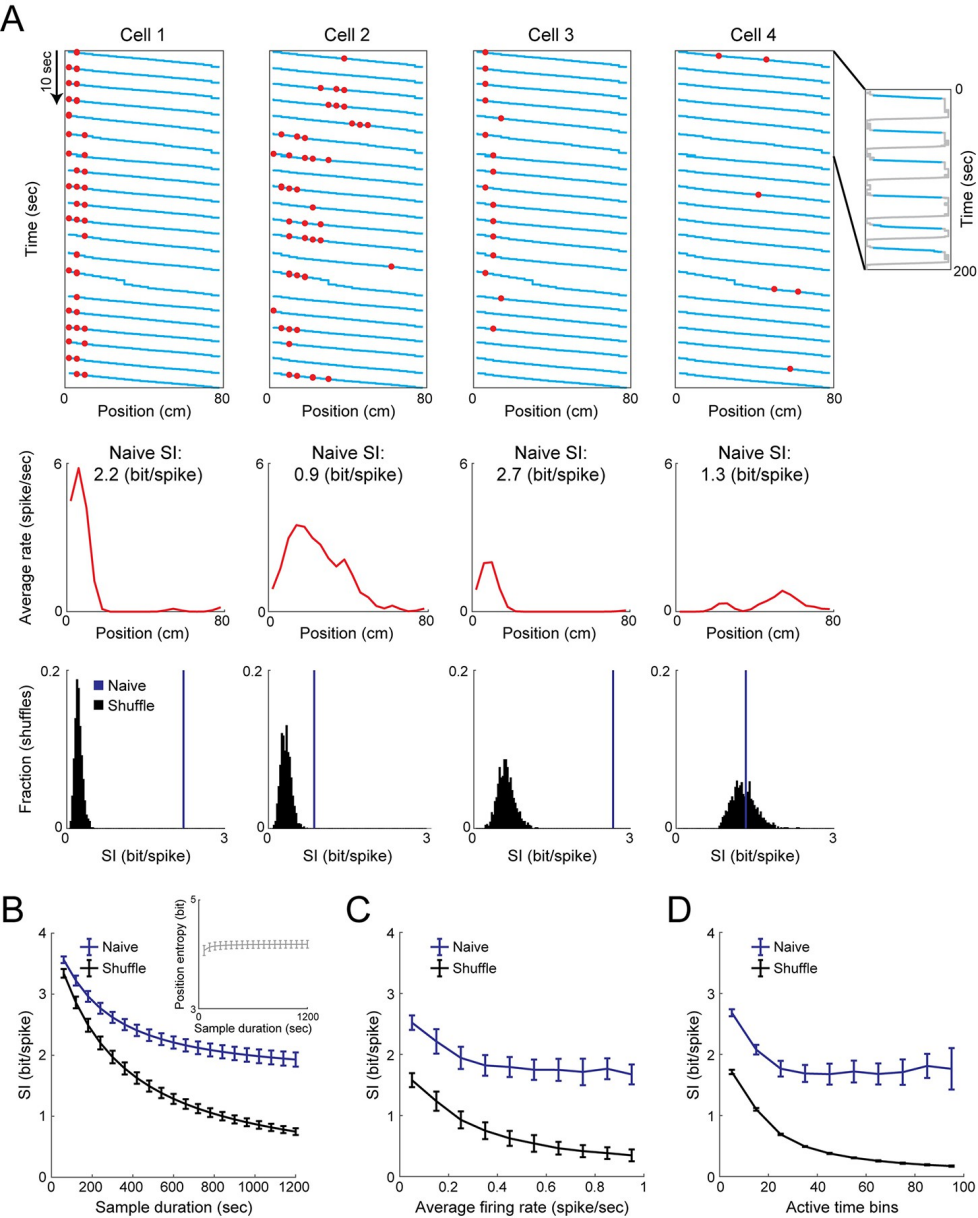
Limited sample sizes and temporally sparse neuronal activity positively bias the naïve calculation of information content. (A) Top: Neuronal activity (red) of four example cells overlaid on the mouse trajectory (cyan) during rightward running epochs in the linear track. Inset, the full mouse trajectory during 200 seconds in the linear track, with the rightward running epochs marked in cyan. Middle: Tuning curves of the same example cells, and their naïve SI expressed in bit/spike. Bottom: The naïve SI (blue) and distribution of shuffle SI (black) of the same cells. Note that the naïve SI of a cell with significant place tuning and high firing rates (cell 2) is lower than that of a cell without significant place tuning and with low firing rates (cell 4). (B) Naïve SI (mean ± SD) as a function of the sample duration for real (blue) and shuffled (black) data. Inset, entropy of the mouse position (mean ± SD) as a function of sample duration. (C-D) The naïve SI (mean ± SD) as a function of the average firing rate (C) or number of active time bins (D) for real (blue) and shuffled (black) data. Data in B-D were averaged across N = 9 mice. For each mouse, SI was averaged across the last four imaging sessions in each of the two environments when they are familiar (a total of 740–4,003 place cells per mouse).

Next, for each place cell, we estimated its spatial tuning curve based on the cell’s estimated firing rate as a function of the mouse position within the linear track (Fig 2A). Then, based on the estimated tuning curves (calculated separately for each running direction), we applied the naïve calculation of SI (*naïve SI*) for each place cell (expressed in bit/spike):

$${SI}_{bit/spike}=\sum_{i}P_{i}\frac{r_{i}}{\bar{r}}{log}_{2}(\frac{r_{i}}{\bar{r}}),$$

where *P*_*i*_ is the probability of the mouse to be in the *i*^th^ spatial bin, *r*_*i*_ is the estimated firing rate in the *i*^th^ spatial bin, and $\bar{r}$ is the overall average estimated firing rate. Place cells were identified by comparing the naïve SI of each cell to the SI in shuffled data (*shuffle SI*), in which the cell’s estimated spike trains were cyclically permuted [15,37–39]. We calculated the average SI across the population of place cells (pooled together from both running directions) for random subsamples of the data (see Methods), and found that it decreases as a function of sample duration (Fig 2B, blue curve), indicating a larger upward bias for more limited sample sizes. Moreover, even in shuffled data, where neuronal activity was independent of the animal’s position, the SI yielded values greater than zero, which also decreased with sample duration (Fig 2B, black curve), resulting in an increase in the fraction of significantly tuned place cells with sample size (S1A–S1C Fig). While a larger fraction of spatially modulated hippocampal place cells were found when the significance test was performed with random permutations rather than cyclic permutations, a similar behavior of the bias in the shuffle SI was observed for the identified place cells in both cases (S1D–S1F Fig). The naïve SI also decreased as a function of the number of track traversals, at a rate similar to that observed as a function of sample duration (S2A Fig). Furthermore, we found only minimal changes in the prior distribution of the animal’s position as a function of sample duration (Figs 2B, inset and S2B), indicating that these results are not due to an inherent bias in the prior distribution generated by our subsampling procedure. By multiplying the SI of each cell by its average firing rate, we obtained the average rate at which information is conveyed (expressed in bit/sec), which exhibited a similar upward bias for limited sample sizes (S3A Fig). Additionally, the naïve calculation of MI (*naïve MI*) exhibited a decrease as a function of sample size (S4A Fig). Together, these results demonstrate an upward bias in the naïve estimation of SI for limited sample sizes, consistent with previous reports on the calculation of MI [10].

Next, we studied how the bias in the naïve calculation of information content is related to the temporal sparsity of the neuronal activity data. Although SI (expressed in bit/spike) should directly reflect the specificity of the tuning curves, we found cells with significant place tuning (Fig 2A, cell 2) that exhibit lower SI than cells without significant place tuning (Fig 2A, cell 4), suggesting that differences in the neuronal activity levels might also influence the naïve SI. Indeed, we observed a decrease in SI as a function of the cells’ average firing rate or the number of active time bins, in both the data (Fig 2C and 2D, blue curves), and shuffled data (Fig 2C and 2D, black curves), consistent with previous reports [15,17]. While the naïve SI expressed in bit/sec and naïve MI increased with the firing rate and number of active time bins, the ratio between the shuffle and naïve information decreased with activity levels, indicating a smaller relative contribution of the bias to the calculated information for the more active cells (S3B–S3F and S4B–S4F Figs). A recent study suggested normalizing the estimated information by calculating a Z-score with respect to the shuffle information, to obtain a more accurate estimation of the information content, which correlates better with the decoding performance [17]. However, the dispersion of the shuffle SI decreases with sample duration, average firing rate, and number of active time bins (S5A–S5C Fig). Consequently, the Z-score of the SI expressed in bit/spike of each cell increases with activity levels (S5D–S5F Fig), similarly to SI expressed in bit/sec, instead of only reflecting the tuning specificity. In any case, since the Z-scored SI values are also highly dependent on sample size, this type of normalization cannot fully alleviate the bias in the naïve SI. Overall, both a limited sample size and temporally sparse neuronal activity positively bias the naïve calculation of information content.

### Methods for correcting the bias in the naïve estimation of information content

**Fig 3:**
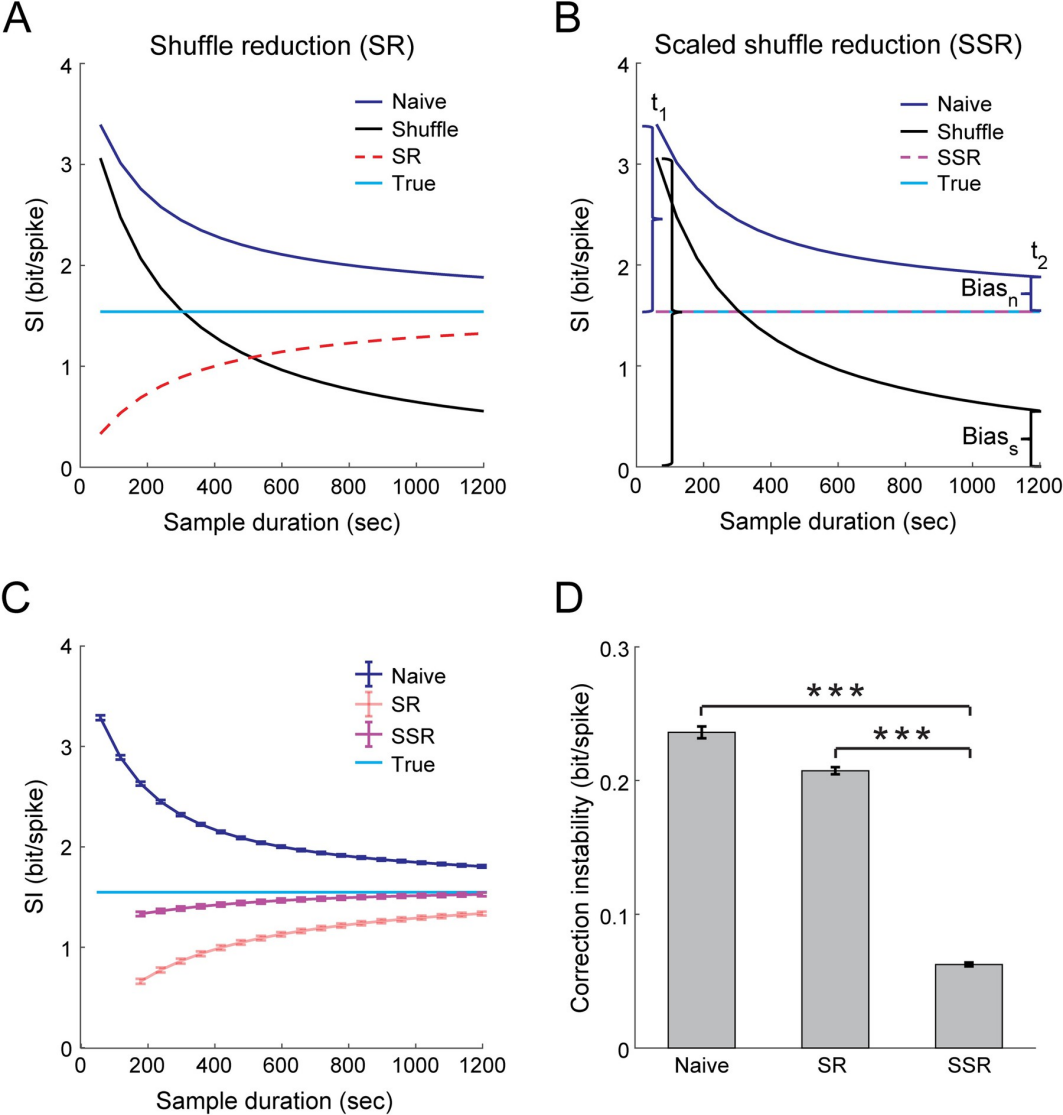
Correcting the upward bias in naïve SI using the SSR method. Validation based on data from simulated place cells with similar tuning properties to those observed in the real hippocampal data, corresponding to 20 minutes of free exploration in the linear track. (A) Demonstration of the shuffle reduction (SR) method. Estimated SI as a function of sample duration for the naïve calculation (blue), shuffle (black), SR (red), and the true SI (cyan). (B) Demonstration of the scaled shuffle reduction (SSR) method. Estimated SI as a function of sample duration for the naïve calculation (blue), shuffle (black), SSR (magenta), and the true SI (cyan). The SSR estimation is obtained by assuming a fixed ratio between the bias in the naïve SI (bias_n_, blue) and the bias in the shuffle SI (bias_s_, black) for two different sample durations, and using this bias ratio to subtract from the naïve SI a scaled version of the shuffle SI. Data in A-B show the mean across 100 cells from one example simulation. (C) Estimated SI (mean ± SEM) as a function of sample duration for the naïve calculation (blue), SR (red), SSR (magenta), and the true SI (cyan). (D) Correction instability (mean ± SEM), defined as the standard deviation of the estimated SI across sample durations, for the naïve calculation, SR, and SSR. SSR is more stable than the naïve calculation (matched-pairs two-sided t-test_(8)_, t = 51.5, p = 2.2·10^−11^) and SR (matched-pairs two-sided t-test_(8)_, t = 54.5, p = 1.4·10^−11^). Data in C-D were averaged across N = 9 simulations. Each simulation corresponds to behavioral data from a different mouse and consists of 100 simulated place cells. ***p < 0.001.

Evaluating methods for correcting the upward bias in the naïve calculation of information content requires data with known ground-truth information values. Therefore, we used the mice trajectories from our experiment and simulated corresponding neuronal activity data of spatially modulated cells with Poisson firing statistics and spatial tuning properties that match the real data (see Methods). The true SI was obtained by applying the naïve calculation to the ground-truth tuning curves. Applying the same calculation to the tuning curves estimated from the activity of the simulated cells yielded a higher estimation than the true SI (Fig 3A). This upward bias decreased with sample size (Fig 3A, blue versus cyan curves), and a similar trend was observed in the shuffle SI (Fig 3A, black curve). Consistent with the indication of a rate-dependent bias in our real data and in previous reports [15,17], the simulations confirmed that the bias in the naïve SI decreases as a function of the average firing rate and active time bins (S6 Fig).

To correct this upward bias, we first tried to use the bias in the shuffle SI (which is the deviation of the shuffle SI from zero) as a direct estimation of the bias, subtracting the shuffle SI of each cell from the naïve SI (shuffle reduction, *SR*) [10,28,33,40]:

$${\hat{SI}}_{SR}=SI_{naive}-SI_{shuffle},$$

where ${\hat{SI}}_{SR}$ is the estimated SI based on the SR method. However, for small sample sizes, the shuffle SI was almost equal to the naïve SI, resulting in a strong overcorrection by the SR method (Fig 3A, red curve). This overestimation of the bias is due to the fact that the shuffled activity covers more spatial bins compared to the activity of spatially modulated cells, requiring even larger sample sizes to yield an unbiased estimation of the shuffle information content [41]. Thus, the ratio between the bias in the naïve SI and the one in the shuffle SI for a given cell should depend on the specificity of its tuning curve. Indeed, we found that there is a cell-specific ratio between the bias in the naïve SI and the shuffle SI (*bias ratio*), which decreases with tuning specificity but not with sample size (S7 Fig). Consistent with the decrease in the bias ratio with tuning specificity, we also found that the bias in the naïve SI itself decreases as a function of the true SI (S8A Fig). Assuming a cell-specific bias ratio that is fixed between two different sample durations, *t*_*1*_ and *t*_*2*_, we obtained:

$$\frac{SI_{naive}(t_{1})-SI_{true}}{SI_{shuffle}(t_{1})}=\frac{SI_{naive}(t_{2})-SI_{true}}{SI_{shuffle}(t_{2})},$$

where *SI*_*true*_ is the true SI, and *SI*_*naive*_*(t)* and *SI*_*shuffle*_*(t)* are the naïvely calculated SI and average SI across all shuffles, respectively, for a subsample duration *t*. *t*_*1*_ is a subsample duration and *t*_*2*_ is the full sample duration. After rearranging the equation and isolating the true SI, we obtained:

$$SI_{true}=SI_{naive}(t_{2})-SI_{shuffle}(t_{2})\frac{SI_{naive}(t_{1})-SI_{naive}(t_{2})}{SI_{shuffle}(t_{1})-{SI}_{shuffle}(t_{2})}.$$

Therefore, we can now use this formula to estimate the true SI by calculating the bias ratio for each cell and subtracting from the naïve SI a scaled version of the shuffle SI (scaled shuffle reduction, *SSR*; Fig 3B):

$${\hat{SI}}_{SSR}=SI_{naive}(t_{2})-SI_{shuffle}(t_{2})\frac{SI_{naive}(t_{1})-SI_{naive}(t_{2})}{SI_{shuffle}(t_{1})-{SI}_{shuffle}(t_{2})},$$

where ${\hat{SI}}_{SSR}$ is the estimated SI based on the SSR method. We found that the SSR method was more accurate and converged to the true SI for shorter sample durations than the SR method (Fig 3C). Although both SR and SSR may overcorrect the bias, SSR required < 6 minutes of data to reach 90% (and < 11 minutes to reach 95%) of the true SI, while SR reached only 86% of the true SI based on the full 20-minutes session length. The higher accuracy of the SSR method over SR was maintained across a wide range of different *t*_*1*_ subsample durations (S9 Fig). The improved estimation was also obtained when SI was expressed in bit/sec (S10A Fig) or for the calculation of MI (S11A Fig), demonstrating the general applicability of the SSR method. To quantify the robustness of SSR to limited sample sizes, we calculated the standard deviation in the bias-corrected SI across different sample durations (Fig 3D). This analysis confirmed that SSR yielded a more consistent estimation compared to SR over a wide range of sample durations. Since these estimators are asymptotically unbiased (i.e., they reach the true value at infinite sample sizes), the SSR method’s convergence to the true SI for shorter sample durations than SR is another indication of its superior estimation quality.

**Fig 4:**
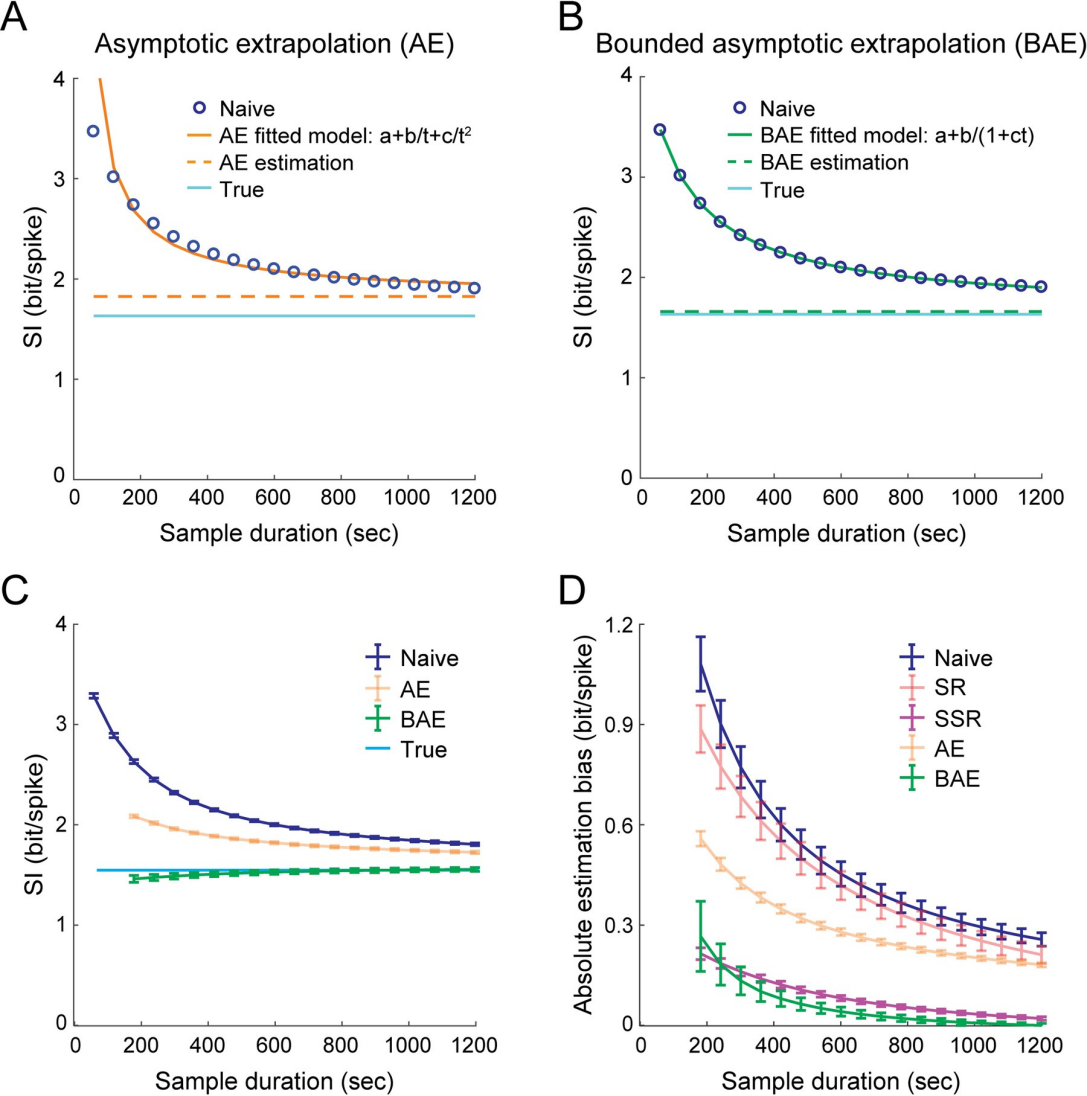
Correcting the upward bias in the naïve SI using the BAE method. (A) Demonstration of the asymptotic extrapolation (AE) method. Using an unbounded function of the form *a+b/t+c/t*^*2*^ (solid orange curve) to fit the naïve SI as a function of sample duration (blue) yields an inaccurate estimation (dashed orange curve) of the true SI (cyan). (B) Demonstration of the bounded asymptotic extrapolation (BAE) method. Using a bounded function of the form *a+b/(1+ct)* (solid green curve) to fit the naïve SI as a function of sample duration (blue) yields an accurate estimation (dashed green curve) of the true SI (cyan). The extrapolation of the fitted curve to an infinite sample duration is the estimated SI. Data in A-B show the mean across 100 cells from one example simulation. (C) Estimated SI (mean ± SEM) as a function of the sample duration for the naïve calculation (blue), AE (orange), BAE (green), and the true SI (cyan). (D) Absolute bias in the estimated SI (mean ± SEM) as a function of sample duration for the naïve calculation (blue), SR (red), SSR (magenta), AE (orange) and BAE (green). Data in C-D were averaged across N = 9 simulations. Each simulation corresponds to behavioral data from a different mouse and consists of 100 simulated place cells. ***p < 0.001.

Previous work corrected the bias by fitting a curve to the naïve MI as a function of sample size based on an analytical derivation of the bias at the asymptotic sampling regime [11], and using the extrapolation of the fitted curve to infinite amount of data as an estimation of the true MI. The function used for this asymptotic extrapolation (*AE*) method is of the form *MI*_*naive*_*(t) = a+b/t+c/t*^*2*^, where *t* is the sample duration, *a* is a free parameter representing the true MI, and *b* and *c* are free parameters that represent how the bias changes with sample duration [30]. Applying this procedure, the estimated SI based on the AE method is:

$${\hat{SI}}_{AE}=\underset{a}{\mathrm{argmin}}\;L(a+\frac{b}{t}+\frac{c}{t^{2}};SI_{naive}(t)),$$

where *L* is the loss (squared errors) for fitting *SI*_*naive*_*(t)* with the defined function. However, since the analytical derivation of the bias was based on an asymptotic expansion, it applies only to large sample sizes [11]. In practice, the naïve information is bounded, and therefore such an unbounded function form, which goes to infinity as *t* approaches zero, fails to accurately fit the bias for short sample durations (Fig 4A). This fit inaccuracy leads to an overestimation of the true information. Therefore, we sought a similar but bounded function that can accurately fit the bias in small sample durations while coinciding with the analytical derivation of the bias in large sample durations. To this end, we fitted the naïve SI using a function of the form *SI*_*naive*_*(t) = a+b/(1+ct)* and extrapolated the fitted bounded curve to infinite amount of data as an estimation of the true SI (bounded asymptotic extrapolation, *BAE*; Fig 4B). Thus, the estimated SI based on the BAE method is:

$${\hat{SI}}_{BAE}=\underset{a}{\mathrm{argmin}}\;L(a+\frac{b}{1+ct};\;SI_{naive}(t))$$

Indeed, BAE yielded a more accurate estimation of the true SI compared to AE (Fig 4C). The higher accuracy of the BAE method was also obtained for the estimation of SI expressed in bit/sec (S10B–S10D Fig) and for the estimation of MI (S11B–S11D Fig). In addition, we found that the decay rate of the bias with sample duration (approximated by the parameter *c* in the BAE fitted curve) was lower for cells with lower activity levels (S12 Fig). This result is consistent with the higher bias observed for cells with lower activity levels (Figs 2C, 2D, and S6) and suggests that the deviation of AE from BAE should be higher for those cells. Limiting the range of the subsample durations used for the AE method by excluding the shorter subsamples improved its estimation accuracy (S13 Fig). Nevertheless, BAE was more robust to changes in the minimal subsample duration *t*_*min*_, and it outperformed AE across the entire range of *t*_*min*_ values. Alternative bounded and monotonically decreasing function types with the same number of free parameters did not yield an accurate fit of the naïve SI or an accurate estimation of the true SI (S14 Fig), indicating that the BAE’s accurate fit of the bias is not trivial. Overall, the SSR and BAE methods yielded a considerably less biased estimation of the true information compared to SR and AE (Fig 4D).

### Validating the accuracy of the methods at the level of individual neurons based on simulated data and Ca^2+^ imaging data

**Fig 5:**
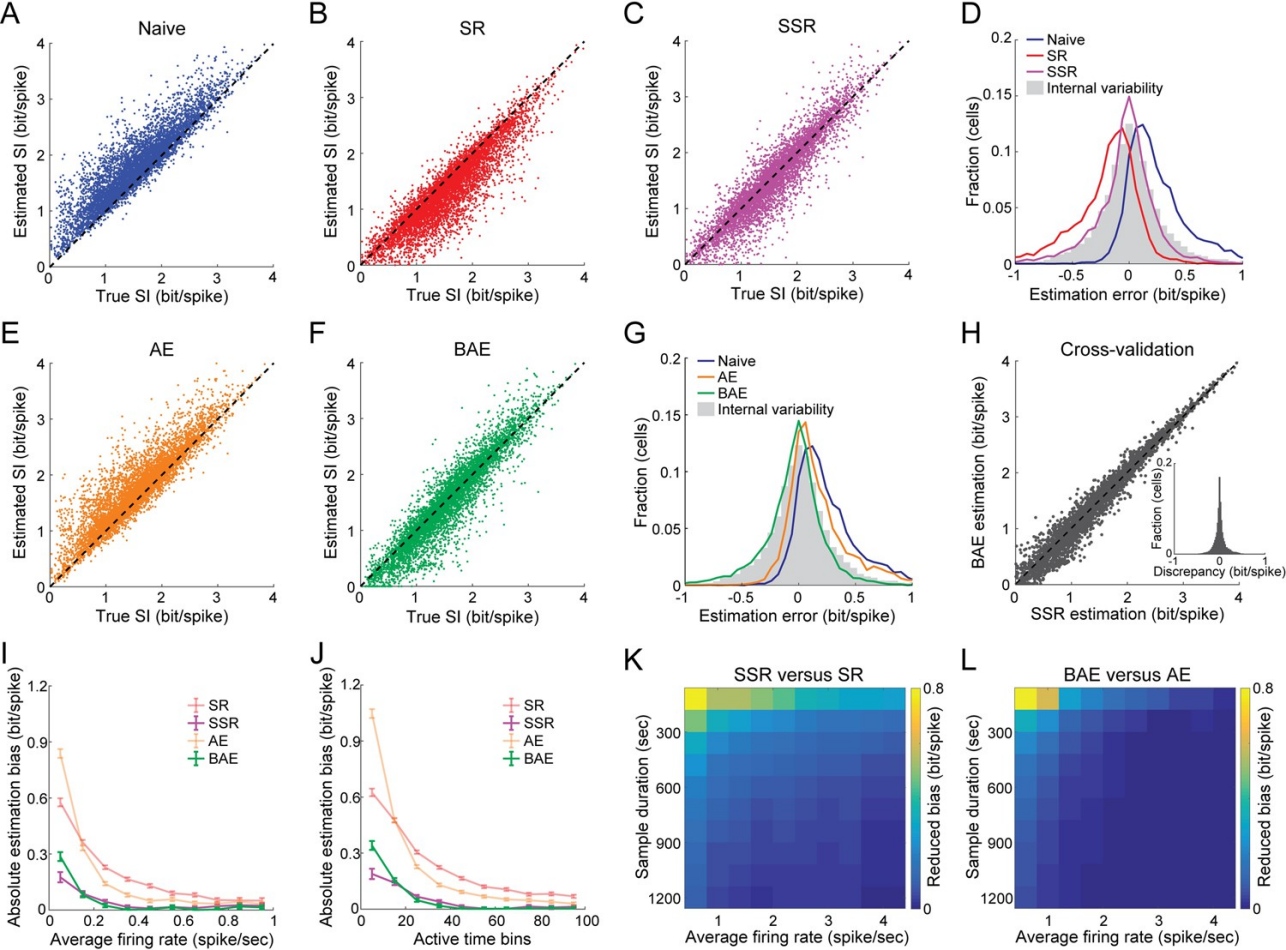
Validating the accuracy of the different bias-correction methods in estimating the true SI of individual simulated neurons. (A) The naïve SI versus the true SI for simulated place cells with similar tuning properties to those observed in the hippocampal data. (B-C) The estimated SI of individual simulated neurons versus their true SI for the SR (B) and SSR (C) methods. (D) Distribution of the estimation errors of individual neurons for the naïve calculation (blue), SR (red), SSR (magenta), and the deviations between the naïve SI of each cell across different realizations (gray). (E-G) Same as in B-D but for AE (orange) and BAE (green). (H) Estimated SI of the same simulated neurons for the BAE method versus the SSR method. Inset, discrepancy between the estimated SI using SSR versus BAE. (I-J) Absolute estimation bias (mean ± SEM) as a function of the cells’ average firing rates (I) or number of active time bins (J) for SR (red), SSR (magenta), BAE (green) and AE (orange). Data were averaged across N = 9 simulations. (K-L) Differences in the absolute estimation bias between SR and SSR (K) and between AE and BAE (L) as a function of both the cells’ average firing rates and the sample duration. These results show that SSR and BAE yield a smaller bias than SR and AE, especially when the firing rates are low or the sample durations are short, indicating they are more robust. Data pooled from N = 9 simulations. Each simulation corresponds to behavioral data from a different mouse and consists of 100 simulated place cells.

We next tested the accuracy of the methods in correcting the bias in the naïve estimation of information content for individual simulated neurons. As expected, the naïve SI exhibited a large upward bias (0.26 ± 0.28 bit/spike [mean ± SD] deviation from the true SI; Fig 5A). While SR showed a strong overcorrection (-0.21 ± 0.29 bit/spike [mean ± SD] deviation from the true SI; Fig 5B), and AE resulted in an insufficient correction (0.18 ± 0.28 bit/spike [mean ± SD] deviation from the true SI; Fig 5E), SSR and BAE yielded a more accurate estimation of the true SI (-0.04 ± 0.27 bit/spike and -0.05 ± 0.27 bit/spike [mean ± SD] deviation from the true SI for SSR and BAE, respectively; Fig 5C, 5D, 5F and 5G). Notably, the deviations of the SSR and BAE methods from the true SI were not larger than the internal variability in the SI of the same simulated cells across different realizations (0.01 ± 0.29 bit/spike [mean ± SD] discrepancy between realizations; Fig 5D and 5G, gray bars), indicating that our methods reduce the bias in the estimation of SI without increasing the estimation variance. Furthermore, there was close agreement in the estimated SI of each cell across our two independent methods (0.006 ± 0.13 bit/spike [mean ± SD] discrepancy between SSR and BAE; Fig 5H), demonstrating the utility of using both methods to cross-validate their accuracy. The advantage of the SSR and BAE methods over SR or AE was most pronounced for cells with low firing rates (Fig 5I and 5J), or under short sample durations (Fig 5K and 5L). Moreover, while SR yielded larger biases for cells with higher tuning specificity and AE generated larger biases for cells with lower tuning specificity, SSR and BAE exhibited smaller biases across a wide range of SI values (S8B Fig). We also tested our methods on data parameters that reside outside the regimes for which the SI is derived from the MI (see Methods). As expected, the SI expressed in bit/sec did not provide an accurate approximation of the true MI rate, with the discrepancy between them increasing both with the size of the time bin and with the deviation of the firing rate distributions from Poisson statistics (S15A and S15B Fig). Focusing on MI in this analysis, we found that the bias in the naïve MI increased with the variability in the firing rate distributions (S15C and S15D Fig). Nonetheless, our methods outperformed previous methods, yielding a more accurate estimation of the true MI for different levels of variability (S15E and S15F Fig). Overall, our methods allowed an unbiased, accurate estimation of the information content of individual simulated neurons with temporally sparse activity and of limited sample sizes over a wide range of parameter regimes.

**Fig 6:**
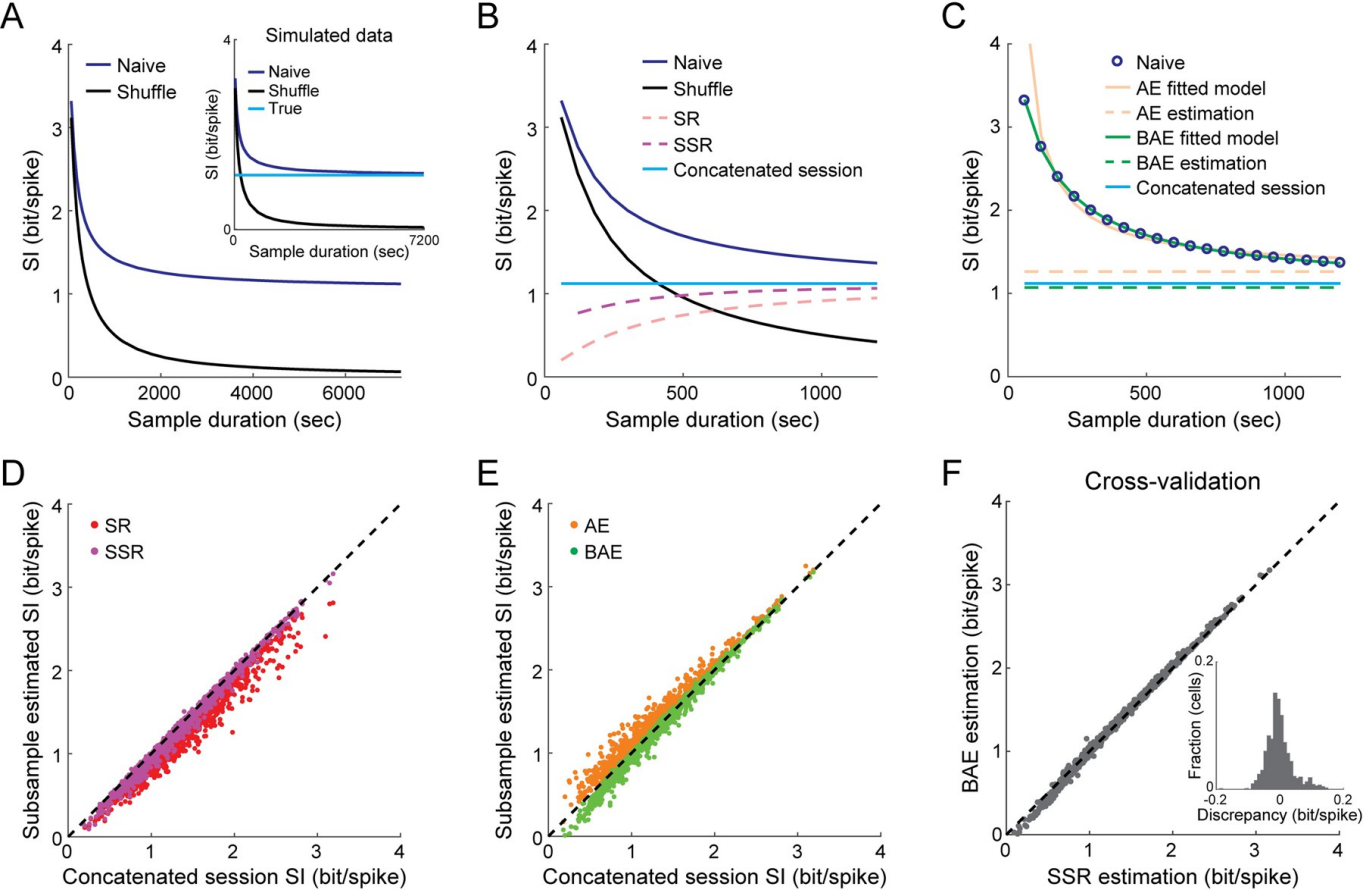
Validating the bias-correction methods on real data by tracking the same neurons across multiple sessions. (A) Naïve SI as a function of the sampling duration for a 2-hour-long concatenated session, for real (blue) and shuffled (black) data. Inset, the same for simulated data, with the true SI shown in cyan. (B) Estimated SI as a function of sample duration from a 20-minute-long subsample for the naïve calculation (blue), shuffle (black), SR (red), SSR (magenta), and the full 2-hour-long concatenated session SI (cyan). (C) Estimated SI from a 20-minute-long subsample for the naïve calculation (blue), AE (orange), BAE (green), and the full 2-hour-long concatenated session (cyan). Data in A-C show the mean across the recorded cells from one example mouse. (D) Bias-corrected SI for individual neurons using SR (red) and SSR (magenta) on 20-minute subsamples versus the naïve SI of the full concatenated 2-hour-long session. (E) Bias-corrected SI for individual neurons using AE (orange) and BAE (green) on 20-minute subsamples versus the naïve SI of the full concatenated 2-hour-long session. (F) Estimated SI of the same neurons for the BAE method versus the SSR method. Inset, discrepancy between the estimated SI using SSR versus BAE. Data in D-F are for 947 place cells that were pooled from N = 9 mice (25–254 cells per mouse), tracked across 6 imaging sessions in the same environment and found to be active in at least 5 of those sessions.

Next, we sought to validate the performance of our bias-correction methods on real neuronal data. Since the ground-truth information content of real data is unknown, we used the ability to track the same neurons across multiple sessions in Ca^2+^ imaging data [4] to validate the methods. Specifically, we concatenated six 20-minute-long recording sessions into a single 2-hour-long session (we excluded the first two sessions, when the environment was novel) and calculated the SI based on the entire concatenated session (Fig 6A). As expected, the bias in the naïve SI was very small for the 2-hour-long session, as demonstrated by the shuffle SI approaching zero. Notably, for 2-hours of simulated data with matching firing rates and tuning statistics, the naïve SI converged to the true SI and the shuffle SI approached zero, confirming that both the subsampling and shuffling procedures do not generate any systematic biases (Fig 6A, inset). Therefore, we used the naïve SI of the concatenated session as a proxy for the ground-truth information and tested the accuracy of the bias-correction methods on a 20-minute subsample of the concatenated session (Fig 6B and 6C). Both the SSR and BAE methods recovered the average naïve SI (across all place cells) of the full concatenated session more accurately than the SR or AE methods, consistent with the results obtained for the simulated data. We then tested the accuracy of the bias-correction methods for real data at the level of individual neurons. The SSR and BAE methods yielded similar SI values to the naïve SI of the concatenated session (Fig 6D and 6E; -0.05 ± 0.05 bit/spike [mean ± SD] and -0.04 ± 0.06 bit/spike [mean ± SD] deviation from the concatenated session for SSR and BAE, respectively). The small negative deviations of these two methods from the naïve SI suggest that a small bias may still exist even for a concatenated 2-hour-long session. In contrast, SR yielded much lower SI values (-0.17 ± 0.1 bit/spike [mean ± SD] deviation from the concatenated session) and AE yielded considerably higher SI values than the concatenated session naïve SI (0.09 ± 0.09 bit/spike [mean ± SD] deviation from the concatenated session). Notably, there was strong agreement in the estimated SI values of individual neurons between SSR and BAE (Fig 6F; 0.002 ± 0.04 bit/spike [mean ± SD] discrepancy between the two methods), cross-validating the two bias-correction methods. Together, these results demonstrate the internal consistency of the SSR and BAE methods, further validating their accuracy when applied to real neuronal data.

### Demonstrating the general applicability of the bias-correction methods to different brain regions and encoded variables

**Fig 7:**
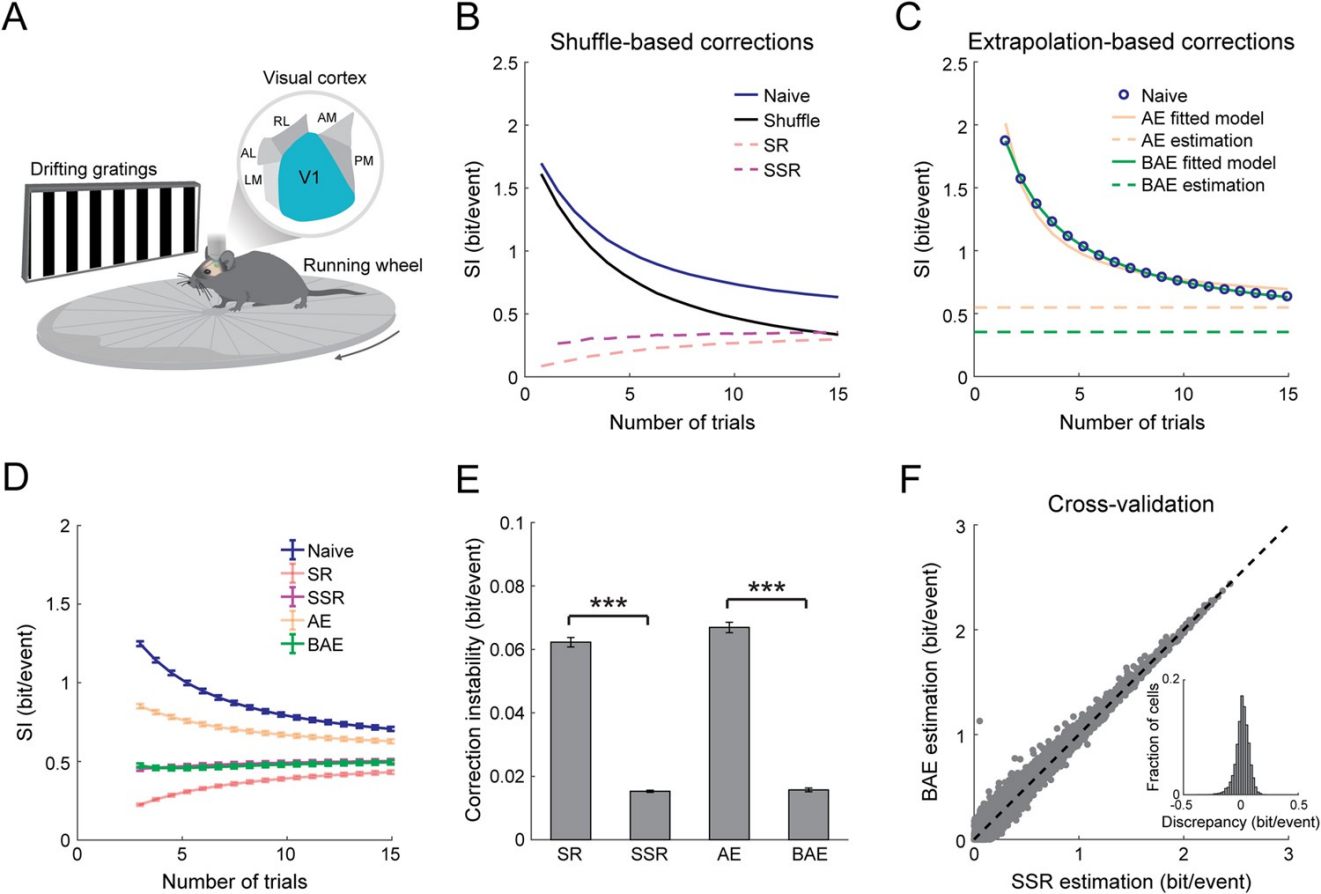
Bias-correction methods are applicable to quantifying information in recordings of neuronal activity from the visual cortex. (A) Ca^2+^ imaging in the primary visual cortex (V1) of mice during the repeated presentation of drifting gratings. Data taken from the Allen Brain Observatory (see Methods) [42]. (B) Quantification of information carried about the direction of movement and temporal frequency of drifting gratings. Estimated SI as a function of the number of trials for the naïve calculation (blue), shuffle (black), SR (red), and SSR (magenta). (C) Estimated SI for the naïve calculation (blue), AE (orange), and BAE (green). Data in B-C show the mean across recorded cells from one example mouse. (D) Estimated SI (mean ± SEM) as a function of the number of trials obtained by the naïve calculation (blue), SR (red), SSR (magenta), AE (orange), and BAE (green). (E) The correction instability (mean ± SEM), defined as the standard deviation of the estimated SI across numbers of trials, for each method. SSR is more stable than SR (matched-pairs two-sided t-test_(93)_, t = 33.8, p = 5.4·10^−54^) and BAE is more stable than AE (matched-pairs two-sided t-test_(93)_, t = 25.3, p = 1.7·10^−43^). Data in D-E were averaged across N = 94 mice. (F) Estimated SI for the same individual neurons (10,225 cells pooled from 94 mice) for the BAE method versus the SSR method. Inset, discrepancy between the estimated SI using SSR versus BAE. ***p < 0.001.

To test the general applicability of our methods to the quantification of the encoding of different variables in other brain areas, we used two-photon Ca^2+^ imaging data from the mouse primary visual cortex (V1) during the repeated presentation of drifting gratings (Fig 7A; publicly available data from the Allen Brain Observatory [42]). Based on the ΔF/F_0_ traces, we set the values of the frames that passed the event-detection criteria to 1 and the rest to 0 (see Methods) [43]. Then, we estimated the information carried by the extracted neuronal activity about the direction and temporal frequency of the movement of the gratings. Similar to the hippocampal data, the naïve SI in V1 neurons decreased with the number of trials for real data and for shuffled data (Fig 7B). We next applied the SSR and BAE methods to the naïve SI and compared their performance to that of the SR and AE methods (Fig 7B and 7C). SSR and BAE yielded similar estimated SI even for small numbers of trials, while SR and AE generated substantially lower and higher estimations, respectively (Fig 7D). The standard deviation in the bias-corrected SI across different numbers of trials revealed that SSR and BAE yielded a more consistent estimation compared to SR and AE (Fig 7E). Finally, the estimated SI values of individual neurons were similar between SSR and BAE (Fig 7F; 0.013 ± 0.06 bit/event [mean ± SD] discrepancy between the two methods), cross-validating their accuracy. Thus, the improved performance of our methods over previous ones obtained for hippocampal place coding are recapitulated for the encoding of information about drifting gratings in the primary visual cortex. Taken together, our results demonstrate the generality of our methods for the bias-free estimation of information content and their applicability across different brain regions and distinct encoded variables.

### Estimating the precision of the neural code by applying bias-correction methods

**Fig 8:**
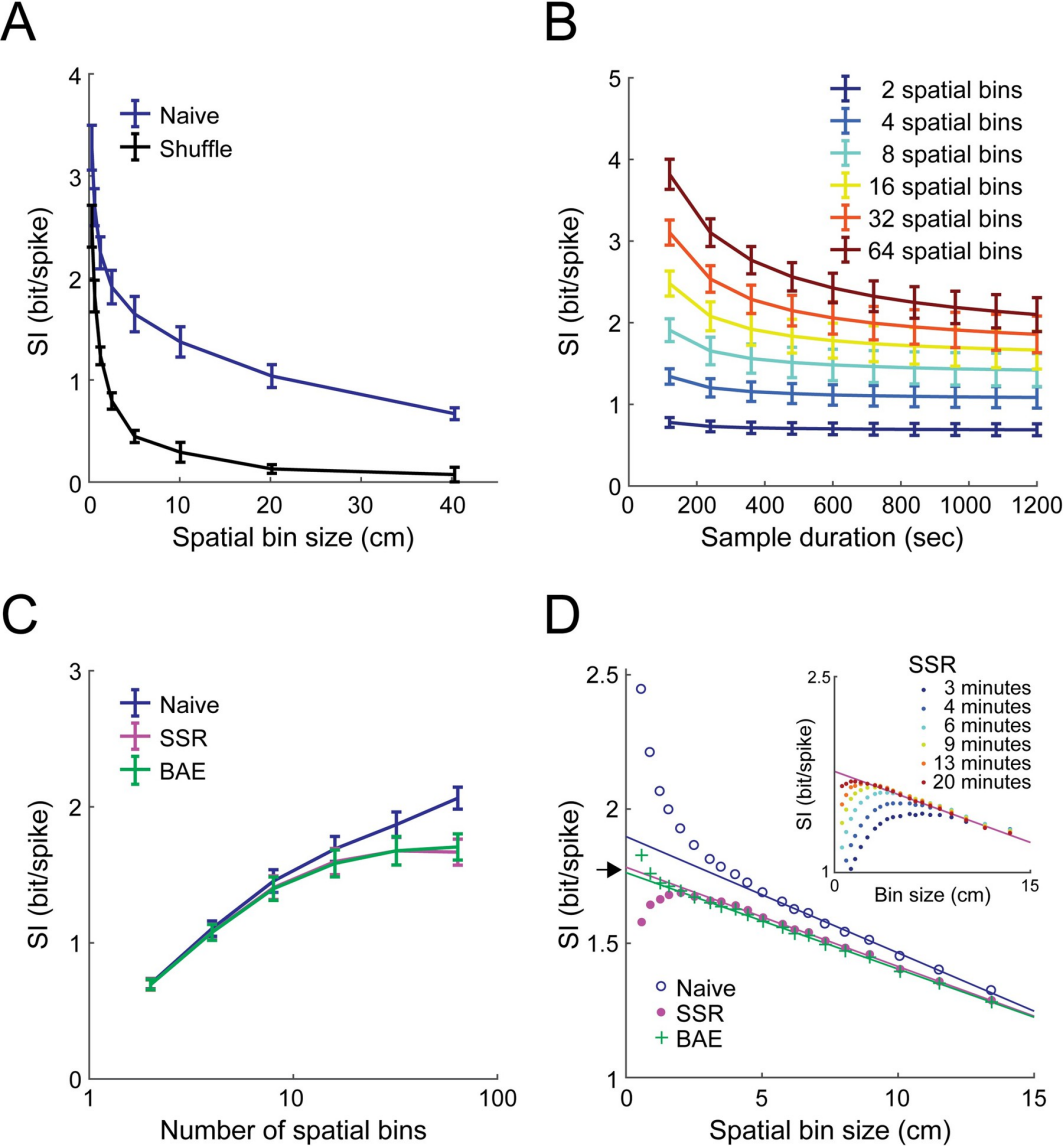
Bias-correction methods allow the estimation of spatial information content independently of the specific choice of spatial binning. (A) Estimated SI (mean ± SEM) as a function of the spatial bin size for the naïve calculation (blue) and shuffle (black). (B) The naïve SI (mean ± SEM) as a function of the sample duration for different numbers of spatial bins. (C) Estimated SI (mean ± SEM) as a function of the number of spatial bins (log-scale) for the naïve calculation (blue), SSR (magenta), and BAE (green). While for the naïve calculation, the SI increases with the number of spatial bins, the SSR and BAE methods reach a stable estimation for a sufficient number of bins. (D) Estimated SI (mean) as a function of the bin size for the naïve calculation (blue), SSR (magenta), and BAE (green). The full-resolution SI (independent of bin size) was estimated using a linear extrapolation to bin size = 0 (indicated by the black arrow). Inset, the same analysis for different sample durations. Data were averaged across N = 9 mice. For each mouse, SI was averaged across the last four imaging sessions in each of the two environments when they were familiar.

A major issue with calculating information theoretic quantities for continuous variables is the requirement for an arbitrary discretization of the set of values the variable can exhibit. For instance, estimating the spatial information of place cells requires dividing the continuous variable of position within the environment into discrete spatial bins, with the arbitrary choice of the discrete set of bins affecting the calculated information. We therefore tested whether our bias-correction methods accurately estimate the true spatial information of hippocampal place cells regardless of the spatial bin size. First, we calculated the naïve and shuffle SI for different spatial binning resolutions, which revealed that both of them monotonically increased as the spatial bin size decreased (Fig 8A and 8B). This result is expected, since naïvely estimating spatial information with higher resolution (more spatial bins) should lead to larger biases [11]. Then, we applied the different bias-correction methods to the data and estimated the true SI as a function of the number of spatial bins (Fig 8C). In contrast to the naïve SI, the bias-corrected SI values estimated using SSR or BAE reached a plateau (at similar values) and did not substantially increase when the position was divided into more than 32 spatial bins (bin size ≈ 2.5 cm). Furthermore, this analysis exposed a linear relationship between the bias-corrected SI and bin size in a wide range of sizes (2 cm < bin size < 13 cm), until reaching too small spatial bins, which precluded a reliable bias correction (Fig 8D). Thus, this type of analysis can be used to determine the maximal discretization resolution for reliably quantifying the encoding properties of a continuous variable in a given data set. Following previous work [30], we performed a linear regression between SI and bin size and extrapolated the SI to bin size = 0. The resultant estimate of the average full-resolution (non-discretized) spatial information of hippocampal place cells was 1.78 ± 0.01 bit/spike (mean ± SEM) or 1.76 ± 0.01 bit/spike (mean ± SEM), according to the SSR and BAE methods, respectively. This analysis also revealed that using 2 cm spatial bins is sufficient to extract more than 95% (or 4 cm for 90%) of the full-resolution spatial information in our experiment. Note that when using longer sample durations, the linear relationship between the bias-corrected SI and bin size is maintained for smaller bin sizes approaching zero (Fig 8D, inset). When the same procedure was applied to the naïve SI, we identified a linear relationship between SI and bin size in a narrower range of sizes (5 cm < bin size < 13 cm), and obtained a positively biased estimation of 1.90 ± 0.02 bit/spike (mean ± SEM) for the full-resolution spatial information. Overall, our bias-correction methods can be used to overcome the dependence on assigning arbitrary discrete values to continuous variables when estimating information content.

### Bias correction facilitates the study of neural code properties and their evolution over time

**Fig 9:**
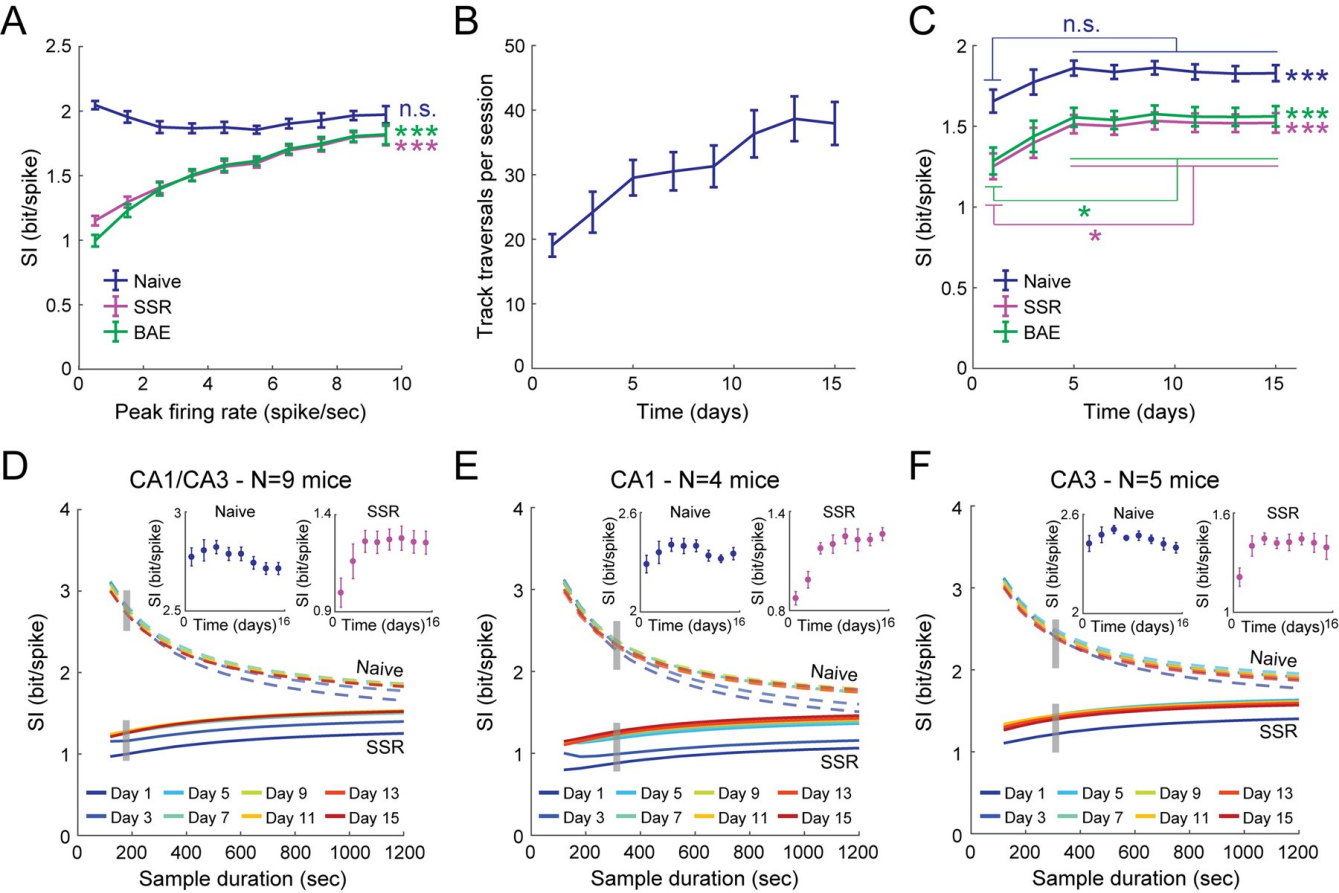
A bias-free estimation of spatial information uncovers hippocampal neuronal tuning properties that may be masked by the bias in the naïve estimation. (A) The estimated SI (mean ± SEM) as a function of the within-field peak firing rates for the naïve calculation (blue), SSR (magenta), and BAE (green). SI was not found to increase with peak rate based on the naïve estimation (linear regression_(10)_, R^2^ = 0.001, slope = -0.001 ± 0.015, p = 0.92), but did increase with peak rate based on SSR (linear regression_(10)_, R^2^ = 0.95, slope = 0.07 ± 0.01, p = 1.8·10^−6^) and BAE (linear regression_(10)_, R^2^ = 0.90, slope = 0.08 ± 0.02, p = 2.8·10^−5^). (B) Average number of track traversals (in each running direction; mean ± SEM) as a function of time in the experiment. Day one is the first exposure to the environment. (C) The estimated SI (mean ± SEM) as a function of time in the experiment for the naïve calculation (blue), SSR (magenta), and BAE (green). SI changed throughout learning for both the naïve estimation (repeated-measures ANOVA_(7)_, F = 7.42, p = 2.7·10^−6^), and the bias-corrected estimations (repeated-measures ANOVA_(7)_, F = 10.32, p = 3.1·10^−8^, and F = 10.10, p = 4.2·10^−8^ for SSR and BAE, respectively). Post hoc pairwise comparison tests revealed that the bias-corrected estimation, but not the naïve, significantly increased with learning. (D-F) Estimated SI (mean) as a function of the sample duration in both hippocampal subfields CA1 and CA3 (D), CA1 only (E), and CA3 only (F) during different learning sessions (colors) for the naïve calculation (dashed curves), and SSR (solid curves). Insets, the SI (mean ± SEM) as a function of time in the experiment was estimated from short subsamples of the data (3 minutes in D and 5 minutes in E-F) using the naïve calculation (left) and SSR (right). Note that even for short sample durations, the bias-corrected estimation clearly reveals an increase in information over time, while the naïve estimation fails to uncover this trend. Data were averaged across N = 9 mice. Data in B-F for each familiarity level (day in the experiment) were averaged across the two environments. For the analyses presented in panels C-F, only place cells with ≥ 10 active time bins in a given session were used. *p < 0.05; ***p < 0.001.

To demonstrate the importance of correcting the upward bias in the naïve calculation of information content for interpreting the neural code, we applied our bias-correction methods to the hippocampal Ca^2+^ imaging data. We first examined the relationship between the within-field peak firing rates of place cells and their spatial information (Fig 9A). Based on the naïve SI, no clear relationship was observed (Fig 9A, blue curve). However, the naïve estimation could suffer from different bias magnitudes across cells with different peak firing rates, precluding a proper comparison between them. Interestingly, estimating the bias-corrected SI with either SSR or BAE revealed that the spatial information of place cells significantly increases as a function of the peak firing rate (Fig 9A, magenta and green curves). These results were recapitulated by subsampling the activity so that all neurons exhibited the same average firing rate (S16A Fig).

Next, we studied the changes in the tuning properties that occur over the course of learning. In this experiment, mice learned to repeatedly traverse linear tracks to obtain water rewards on both ends of the track during multiple 20-minute sessions spanning several weeks. The number of track traversals per session increased with learning, as the mice gradually became familiar with the initially novel linear tracks (Fig 9B). We calculated the average SI for the recorded population of place cells as a function of the session number in a given environment (Fig 9C). However, since the number of track traversals increases with familiarization, the sample sizes are more limited in the earlier sessions, which could result in a larger upward bias in the estimated SI for a novel environment. When based on the full session length, both the naïve and the bias-corrected estimations revealed a significant increase in SI with familiarization, consistent with previous observations of a gradual formation of more informative hippocampal representations [44,45]. However, only the SSR and BAE methods revealed significant differences between pairs of sessions. Importantly, while for shorter sample durations the naïve estimation failed to uncover an increase in information, the bias-corrected estimation clearly exposed the evolution in the spatial information that occurs with learning (Fig 9D). Similar results were obtained by subsampling the data so that the number of track traversals is fixed across the different sessions (S16B and S16C Fig). Furthermore, a comparison between hippocampal subfields CA1 and CA3 revealed that while CA1’s spatial information gradually evolves over multiple days, that of CA3 dramatically increases after the first exposure to the environment and then reaches a plateau (Fig 9E and 9F), consistent with the role of CA3 in rapid memory acquisition of one-time experiences [23,44,46]. Taken together, these analyses demonstrate how a limited sample size and temporally sparse neuronal activity could result in a substantial upward bias in the naïve calculation of information content, which could mask different properties of the neural code. Correcting for this bias can uncover these otherwise overlooked properties and help avoid data misinterpretation.

## Discussion

In this study, we demonstrated that naïvely calculating information content using the Skaggs information index yields a significant upward bias for limited sample sizes, as previously shown for other information theoretic measures such as mutual information [10–13]. In addition, we found that the bias in the naïve calculation of information content is larger when the activity levels are lower. The fact that Ca^2+^ imaging techniques yield temporally sparse neuronal activity traces [3,29,47] emphasizes the need to apply a suitable bias-correction procedure when analyzing such data sets. To address this issue, we developed two distinct methods—SSR and BAE—that generate a bias-free estimation of information content from a limited sample size and temporally sparse neuronal activity. We demonstrated that both methods estimate more accurately than other available methods the information content in Ca^2+^ imaging data recorded from the mouse hippocampus and primary visual cortex. The same improvement was found for the estimation of information in simulated data with matching tuning properties and firing statistics.

Using our bias-correction methods, we revealed differences in spatial information across subsets of hippocampal cells with different within-field firing rates. Our methods also uncovered an increase in hippocampal spatial coding precision over long time periods, in line with previous studies [44,45]. Notably, SSR may overestimate the bias when sample sizes are very limited, which could lead to an artificial increase in the estimated information during learning due to differences in sample sizes across sessions. However, the analysis performed here was based on the full 20-minutes session length, a sample size that we found to be sufficient for SSR to yield a very accurate estimation. Moreover, the strong agreement between SSR and BAE on how information increases during learning provides an important cross-validation, further increasing estimation confidence. Interestingly, our bias-correction methods also allowed us to expose that while information gradually increases in hippocampal CA1 over multiple days, in the CA3 it increases dramatically after the first exposure to the environment and then reaches a plateau, consistent with the role of CA3 in rapid one-trial contextual learning [23,44,46]. The fact that these results would have been missed if the naïve estimation of information content was applied to insufficient sample sizes demonstrates that our bias-correction procedures are necessary to more accurately assess the formation and maintenance of the encoding of task-related variables throughout the brain. Furthermore, both the SSR and BAE methods allowed overcoming the inherent dependency of the estimation on discretization of continuous encoded variables and to uncover the full spatial resolution of hippocampal place coding, independent of the specific choice of spatial binning.

While SI is typically used for hippocampal place cells, as long as the assumption of Poisson firing statistics under which it is derived is met, it is generally applicable to any encoded variable. A few examples outside of place cell research include the quantification of information in head direction cells in the postsubiculum, anterior dorsal thalamic nucleus, and the lateral mammillary nuclei [48,49]; in time cells [50] and sequence cells [51] in the hippocampus; in grid cells in the medial entorhinal cortex [52]; and in cells in the visual cortex that are tuned to location [53]. We tested the generality of our methods by applying them to Ca^2+^ imaging data, to quantify tuning to position in hippocampal place cells and tuning to drifting gratings in the primary visual cortex. Hence, our bias-free estimation of information content can serve as a tool for quantifying the information carried by neuronal activity across different brain regions and about any variable of interest. In addition, we found that our methods can be used to correct the bias in the naïve calculation of MI, including the case of neuronal activity with firing rate distributions that do not follow Poisson statistics. Thus, our methods are also readily applicable to estimating information in a variety of more complex coding schemes such as in the joint activity patterns of groups of neurons [54–56] or in the history dependence of a given neuron on its own spiking activity [40,57].

Different ways to correct the bias in the naïve MI in electrophysiology data have been developed [11,12,30–34]. Here, we built upon principles derived in those studies to correct the bias in the naïve SI when applied to temporally sparse neuronal activity data. Specifically, previous work used the shuffle information as a proxy for the bias and subtracted it from the naïvely calculated information to obtain a significantly less-biased estimation of the information content [10,28,33,40]. However, the shuffle information can yield an overestimation of the bias in the naïve information, resulting in an overcorrection of the SR method [10,12,40,41], which we have found to be exacerbated for small sample sizes or temporally sparse neuronal activity. The SSR method we applied here, on the other hand, relies on the observation that the bias in the shuffle information is larger than the bias in the naïve calculation of information content by a factor that is dependent on the specificity of the tuning curve of each cell [41], but not on sample size. Estimating this bias ratio for each cell allows one to correct the bias in the naïve calculation of information by subtracting from it a scaled version of the shuffle information, yielding a substantially less biased estimation.

Extrapolation methods rely on fitting a curve to the naïvely calculated information as a function of the sample size and then extrapolating it to infinity. An unbounded function, which goes to infinity as the sample size approaches zero, was used in previous work to fit the naïve MI [30]. Therefore, this function may fail to accurately fit the information when the sample size is small. Here, we used a similar but bounded function, which could more accurately fit the bias in the naïve calculation of information content, resulting in a more accurate estimation. While using only larger subsamples for fitting the information improved the performance of the AE method, the BAE method was less sensitive to changes in subsample size and was consistently more accurate. Importantly, the SSR and BAE methods were considerably more robust than the SR and AE methods, which exhibited a strong bias in cases of cells with lower firing rates or short sample durations. Overall, using both the SSR and BAE methods to estimate SI allows one to cross-validate the accuracy of the bias correction and increase the estimation certainty.

While the bias in information theoretic measures due to a limited sample size and temporally sparse neuronal activity may lead to data misinterpretation, several approaches alleviate its effects even without correcting the bias itself. First, it is best if experiments are designed to allow the comparison of data sets with similar sample sizes, to avoid comparing between estimations with different bias magnitudes. This can also be achieved by subsampling the data and diluting the neuronal activity to obtain a fixed sample size and average firing rate. While subsampling does not correct for the bias itself, it at least imposes biases of similar magnitudes across the compared data sets. Another approach is to compare the significance levels of neuronal tuning to a given measured variable instead of comparing the actual values of information content. To measure the significance of tuning, the information content of each cell should be compared to its own shuffles [19,22,28]. For example, a normalization based on Z-scoring the value of the naïve SI with respect to the shuffle SI was suggested as a method to obtain an estimation of SI that is more correlated with decoding performance [17]. However, the variability in the distribution of the shuffle SI decreases with sample size and average firing rate, which results in higher significance levels (or Z-scores) for a given cell, irrespective of the actual specificity of its tuning. Thus, estimating the significance level of each cell and the fraction of significantly tuned place cells in the population also depends on sample size, and therefore subsampling the data to obtain a fixed sample size and average firing rate across cells would be required prior to performing this type of analysis. Importantly, even if subsampling and Z-scoring is applied to the data, correcting for the upward bias in information measures would still be beneficial, as it allows an accurate estimation of the actual values of the true information content. Overall, a bias-free estimation of information improves our ability to accurately analyze and interpret neuronal activity data, enabling an easier comparison of information content across data sets within a given study and even across studies.

## Methods

### Ethics statement

All procedures were approved by the Weizmann Institute Institutional Animal Care and Use Committee (protocol number 01460221–2).

### Animals and surgical procedures

A total of nine C57BL/6 male mice, aged 5–7 months at the start of the imaging (8–12 weeks at the start of viral injections), were used in this study. Four mice were imaged in hippocampal CA1 and five mice were imaged in hippocampal CA3. We used established protocols for *in-vivo* microendoscopy, which allows chronic imaging without any apparent degradation of the imaging quality [58]. We first injected into the CA1 or CA3, under isoflurane anesthesia (1.5–2% volume), 450 nL of viral vector AAV2/5-CaMKIIa-GCaMP6f (~2X10^13^ particles per ml, packed by University of North Carolina Vector Core) [3] at stereotatic coordinates: -1.9 mm anteroposterior, -1.4 mm mediolateral, -1.6 mm dorsoventral from Bregma for CA1, and -1.8 mm anteroposterior, -2.1 mm mediolateral, -2.05 mm dorsoventral from Bregma for CA3. We found in previous work [1,59] that this method yields a stable expression of the GCaMP protein. Mice were then allowed to recover for at least two weeks. Then, we implanted in the CA1 mice a glass guide tube directly above the hippocampus. We used a trephine drill to remove a circular part of the skull. The center of the craniotomy (and accordingly the implanted guide tube) was ~ 0.25 mm lateral and 0.25 mm posterior to the injection site. We removed the dura and cortex above the CA1 by suction with a 29-gauge blunt needle while constantly washing the exposed tissue with sterile phosphate-buffered saline (PBS). We then implanted the optical guide tube with its window just dorsal to, but not within, the CA1 area and sealed the space between the skull and the guide tube using 1.5% agarose in PBS. The exposed skull areas were then sealed with Metabond (Parkell, Edgewood, NY) and dental acrylic. To optically access hippocampal CA3 without damaging CA1, we implanted a microendoscope equipped with a micro-prism (1 mm diameter, 4 mm length). The craniotomy was centered anterolateral to the viral injection site (-1.1 mm anteroposterior, -2.15 mm mediolateral). Then, after removing the dura and cortex above the hippocampus, the micro-prism was inserted using a stereotax so that the posterolateral edge of the prism was located -1.7 mm anteroposterior, -2.55 mm mediolateral, -2.45 mm dorsoventral from Bregma. Similar to the surgery in CA1, the exposed areas of the skull were then sealed with Metabond (Parkell, Edgewood, NY) and dental acrylic. The microendoscope was sealed with silicon for protection until the attachment of a base plate. To perform time-lapse one-photon Ca^2+^ imaging in freely behaving mice using an integrated miniature fluorescence microscope (nVistaHD, Inscopix), we followed a previously established protocol [1]. At least three weeks after guide-tube implantation, mice were imaged under isoflurane anesthesia using a two-photon microscope (Ultima IV, Bruker, Germany) with a GaAsP photomultiplier tube (Hamamatsu model 7422PA-40). We inserted a microendoscope consisting of a single gradient refractive index lens (1 mm diameter, Inscopix) into the guide tube and examined Ca^2+^ indicator expression and tissue health. We selected for further imaging only those mice that exhibited homogenous GCaMP6f expression and appeared to have healthy tissue. Mice with clear signs of tissue compression or patches with missing cells were excluded from the study. We also examined for signs of over-expression, such as nuclear filling of the indicator. Mice in which more than 5% of the cells exhibited nuclear expression of the indicator were also excluded from the study. The selected mice were water-restricted for a few days prior to attaching the microscope’s base plate to avoid changes in the imaging focal plane that may occur during the experiment due to changes in the mice weight. For the CA1 mice, we then affixed the microendoscope within the guide tube using an ultraviolet-curing adhesive (Norland, NOA81, Edmund Optics, Barrington, NJ). Finally, for both the CA1 and CA3 mice, we attached the microscope’s base plate to the dental acrylic cap using a light-cured adhesive (Flow-It ALC, Pentron, Orange, CA).

### Ca^2+^ imaging and experimental timeline

A few days after attaching the microscope’s base plate, mice were habituated to human handling by allowing them to walk on experimenters’ hands. We then began training the water-restricted mice to run back and forth on an elevated, short (56 cm long) linear track with high walls (30 cm high) that was located in a square enclosure within the recording room. Before beginning the Ca^2+^ imaging, we pre-trained the mice for 3 days to run on the short linear track while carrying the head-mounted microscope. Then, we began imaging in a novel straight linear track (environment A, 96 cm long) with low walls (4 cm high). Each imaging day consisted of two 10-minute sessions separated by five minutes during which the mice were placed in a transparent bucket on top of the linear track. Imaging was performed every other day, reaching a total of eight imaging days spanning two weeks. After eight imaging sessions in the straight linear track, eight additional imaging sessions were performed every other day in an L-shaped linear track (Environment B). Three out of the nine mice explored the environments in the opposite order. The environments differed in their geometry and had distinct sets of visual and tactile cues, overhead lights and odor cues. Before the beginning of each imaging session, we wiped the tracks with differently scented paper towels (10% ethanol for environment A and 0.5% acetic acid for environment B). We trained the mice to run back and forth along the track by giving them a measured amount of water sweetened with 4% sucrose. The water reward was dispensed at both ends of the tracks using a custom-made computer-controlled device. To record mouse behavior, we used an overhead camera (DFK 33G445, The Imaging Source, Germany), which we synchronized with the integrated microscope. Ca^2+^ imaging was performed 11–18 weeks after viral injection at 10 Hz (N = 1 mouse) or 20 Hz (N = 8 mice), with a spatial downsampling of a factor of four in each dimension (final pixel size of 2.3 X 2.3 μm).

### Mouse position tracking

We analyzed mouse behavior videos using a custom MATLAB (Mathworks) routine that detects the mouse’s center of mass in each frame. We then used the estimated position to calculate its velocity and applied a smoothing filter (rectangular window of 250 msec) to the calculated velocity.

### Processing Ca^2+^ imaging data

We pre-processed the Ca^2+^ imaging data using commercial software (Mosaic, version 1.1.1b, Inscopix) and custom MATLAB routines as previously described [1,59]. To correct for lateral displacements of the brain, we used a rigid-body registration. This procedure was performed on a high-contrast sub-region of the movies in which the blood vessels were most prominent. To enhance the appearance of the blood vessels that were used as stationary fiducial markers for image registration, we divided each pixel by the corresponding value from a smoothed image. The smoothed image was obtained by applying a Gaussian filter with a radius of 100 μm to the movies. Ca^2+^ dynamics were then extracted from the registered movies using CNMF-e [35], an extension of the constrained non-negative matrix factorization method [60], for one-photon microendoscopic data. This method detects cells in Ca^2+^ imaging data by modeling the videos as a superposition of all neurons’ spatiotemporal activity, plus time-varying background and additive noise. The noise components can compensate for spatial and temporal non-uniform illumination in the Ca^2+^ imaging data. The activity of each neuron is expressed as the outer product of a spatial vector, which represents its spatial footprint, and a temporal vector, which represents its Ca^2+^ trace. In addition, by deconvolving the Ca^2+^ trace using an autoregressive model, the method estimates the underlying spiking activity. We used the estimated underlying spike trains as a proxy for the firing rates of the cells. For each mouse, the same parameters (3 ≤ gsig ≤ 4, gsiz = 8, 0.7 ≤ min_corr ≤ 0.85, 8 ≤ min_pnr ≤ 18) were maintained across all sessions. CNMF-e allowed the detection of hundreds of neurons per imaging session, and yielded the estimated spike train for each neuron.

### Tracking the same neurons across sessions

To identify the same neurons across multiple imaging sessions, we used a probabilistic method for cell registration [4], which estimates the probability of correct registration for each cell in the data set and the overall rates of registration errors. First, the different sessions are aligned to one another by maximizing the cross-correlation of the centroid locations between each session and a reference session (session 1). Then, the distribution of Pearson correlations between the spatial footprints (spatial correlation) of pairs of neighboring cells (maximal distance = 14 μm) across sessions is computed. Next, the distribution of spatial correlation is modeled as a weighted sum of two subpopulations, one corresponding to the same cells and the other to different cells. This allows the estimation of the overall rates of false-positive errors (different cells falsely registered as the same cells) and false-negative errors (the same cells falsely registered as different cells) as a function of the registration threshold (P_same_ = 0.5 was used for all mice), yielding a registration that is optimized to the data set of each mouse.

### The naïve calculation of information content

For place tuning analysis, we considered periods wherein the mouse ran > 1 cm/sec. We divided each track into 24 equal spatial bins (4 cm per bin) and computed the time spent and estimated spike number in each bin. We then computed the tuning curve (spike rate per spatial bin, excluding the last 2 bins at each end of the track) for each neuron by dividing the map of spike numbers by the map of occupancy. We separately considered place fields for the two running directions on the linear track. We then focused only on active cells (≥ 5 active time bins during the running periods of a given session, unless specified otherwise). We applied the Skaggs information index (SI) [15] to the tuning curves of each cell, obtaining the naïve estimation of its information content, expressed in bit/spike:

$${SI}_{bit/spike}=\sum_{i}P_{i}\frac{r_{i}}{\bar{r}}{log}_{2}(\frac{r_{i}}{\bar{r}}),$$

where *P*_*i*_ is the probability of the mouse to be in the *i*^th^ bin (time spent in *i*^th^ bin/total running time); *r*_*i*_ is the estimated firing rate in the *i*^th^ bin; and $\bar{r}$ is the overall average estimated firing rate based on the CNMF-e output. In addition, we multiplied the SI of each cell by its average firing rate, obtaining the naïve estimation of its information content, expressed in bit/sec:

$${SI}_{bit/sec}=\sum_{i}P_{i}r_{i}{log}_{2}(\frac{r_{i}}{\bar{r}}).$$

We then calculated the SI for 1,000 distinct cyclic shuffles, shifting the entire estimated spike train by a different amount of time in each shuffle [15,37–39]. The shuffles were used to obtain the p-value of the calculated SI and determine in which cells, the activity was significantly modulated by the animal’s position. Cells whose SI was higher than that in 95% of their shuffles were considered significant place cells. For each place cell, we also estimated its mutual information (MI) [5]. For this purpose, we discretized the estimated spike trains by setting the values to the closest integer up to 10 spikes/time bin. Then, we applied the naïve calculation of MI (*MI*_*naive*_) to the discretized activity of each cell [16,17], expressed in bits:

$$MI=\sum_{i}\sum_{r}P_{i,r}{log}_{2}\left(\frac{P_{i,r}}{P_{i}P_{r}}\right),$$

where *P*_*i*,*r*_ is the joint probability of the mouse to be in the *i*^th^ spatial bin and the neuron to fire r spikes within a given time bin, *P*_*i*_ is the probability of the mouse to be in the *i*^th^ spatial bin, and *P*_*r*_ is the overall probability of the neuron to fire *r* spikes within a given time bin. For obtaining the MI rate expressed in bit/sec, we divided the MI by the size of the time bin. The time bin was set to 50 msec (or 100 msec) for mice imaged at 20 Hz (or 10 Hz), unless specified otherwise.

### Information as a function of sample duration

Previous work has shown that MI suffers from a significant systematic error (bias) when the amount of data is limited [10,11]. Therefore, we examined how the naïve calculation of SI (*naïve SI*) changes as a function of sample duration. Specifically, we performed random subsampling of the data, using different fractions, ranging between 5–100% of the full data size. Since at small sample sizes, there is higher variability in the calculated information, due to the random subsampling procedure, we increased the statistical power by averaging the calculated information over 500 subsampling repetitions for each subsample size. Similarly, we calculated the naïve SI as a function of the number of track traversals. For this analysis, we randomly subsampled full track traversals and then averaged the calculated information across 500 subsample repetitions with the same number of traversals.

### Shuffle-based bias-correction methods

Since the naïve SI is dependent on the sample duration, we consider *SI*_*naive*_*(t)* to be the sum of two components: 1) the duration-independent true SI (*SI*_*true*_); and 2) the duration-dependent bias term (*bias*_*naive*_*(t)*):

$$SI_{naive}(t)=SI_{true}+{bias}_{naive}(t).$$

Importantly, for an infinite amount of data, the bias approaches zero and the naïve SI converges to the true SI (i.e., asymptotically unbiased).

### Shuffle reduction (SR)

An additional indication of the upward bias in the naïve SI for limited sample sizes is the fact that shuffling neuronal activity data results in non-zero SI values, which increase as the sample size decreases. The most straightforward shuffle-based bias-correction is to use the average value across shuffling repetitions (*shuffle SI*) as a direct estimation of the bias and subtract the shuffle SI from the naïve SI of each cell to obtain the estimated SI based on the SR method [10,17,28,33]:

$${\hat{SI}}_{SR}=SI_{naive}(t)-SI_{shuffle}(t)$$

We obtained shuffled data by randomly distributing the spikes across the different time points within a given imaging session (contrary to the cyclic shuffling used for the spatial modulation significance test described above), which completely breaks the statistical relationship between neuronal activity and the measured variable (e.g., mouse position). We then averaged the shuffle SI calculated for each cell over 500 shuffling repetitions. Since the naïve SI is asymptotically unbiased and the shuffle SI converges to zero for infinite amount of data, SR yields an asymptotically unbiased estimation of the true SI. The shuffles were also used to obtain the Z-score of each cell with respect its shuffles [15,17].

### Scaled shuffle reduction (SSR)

The SR method’s overestimation of the bias is due to the fact that the shuffled activity covers more spatial bins compared to the activity of spatially modulated cells, leading to a larger bias in the calculation of the shuffle information [41]. Thus, the ratio between the bias in the naïve information and the bias in the shuffle information for a given cell depends on its tuning curve. To account for that, we assumed there is a cell-specific ratio between the bias in the naïve SI (*bias*_*naive*_) and the bias in the shuffle SI (*bias*_*shuffle*_) that is independent of the sample size:

$$\frac{{bias}_{naive}(t)}{{bias}_{shuffle}(t)}=\frac{SI_{naive}(t)-{SI}_{true}}{SI_{shuffle}(t)-0}=constant.$$

Assuming a cell-specific bias ratio that is fixed between two different sample durations, *t*_*1*_ and *t*_*2*_, we obtained:

$$\frac{SI_{naive}(t_{1})-{SI}_{true}}{SI_{shuffle}(t_{1})}=\frac{SI_{naive}(t_{2})-{SI}_{true}}{SI_{shuffle}(t_{2})}.$$

After rearranging the equation and isolating the true SI, we obtained:

$$SI_{shuffle}(t_{2})(SI_{naive}(t_{1})-{SI}_{true})=SSI_{shuffle}(t_{1})(SI_{naive}(t_{2})-{SI}_{true})==>$$

$${SI}_{true}=\frac{SI_{shuffle}(t_{1})SI_{naive}(t_{2})-SI_{shuffle}(t_{2})SI_{naive}(t_{1})}{SI_{shuffle}(t_{1})-{SI}_{shuffle}(t_{2})}=\frac{SI_{naive}(t_{2})(SI_{shuffle}(t_{1})-SI_{shuffle}(t_{2}))+SI_{naive}(t_{2})SI_{shuffle}(t_{2})-SI_{shuffle}(t_{2})SI_{naive}(t_{1})}{SI_{shuffle}(t_{1})-SI_{shuffle}(t_{2})}=SI_{naive}(t_{2})-SI_{shuffle}(t_{2})\frac{SI_{naive}(t_{1})-SI_{naive}(t_{2})}{SI_{shuffle}(t_{1})-{SI}_{shuffle}(t_{2})}$$

Therefore, we can now use this formula to estimate the true SI by calculating the bias ratio for each cell and subtracting from the naïve SI a scaled version of the shuffle SI:

$${\hat{SI}}_{SSR}=SI_{naive}(t_{2})-SI_{shuffle}(t_{2})\frac{SI_{naive}(t_{1})-SI_{naive}(t_{2})}{SI_{shuffle}(t_{1})-{SI}_{shuffle}(t_{2})},$$

where ${\hat{SI}}_{SSR}$ is the estimated SI based on the SSR method.

### Extrapolation-based bias-correction methods

An extrapolation method was proposed in previous work as a means to correct the bias in the naïve MI [30]. This method fits a curve to the naïve MI as a function of the sample size and extrapolates the fitted curve to infinity, where the bias approaches zero. Since the bias term in the naïve MI can be analytically derived [11], this derivation allows one to determine the type of function that should be used to fit the data.

### Asymptotic extrapolation (AE)

Based on the analytical derivation of the bias term at the asymptotic sampling regime [11], previous work fitted the naïve MI as a function of sample size using a function of the form [30]:

$$MI_{naive}(t)=a+\frac{b}{t}+\frac{c}{t^{2}},$$

where *a* is a free parameter that represents the estimated value for the true MI, *t* is the sample duration, and *b* and *c* are free parameters for fitting the bias term. Applying this procedure, the estimated SI based on the AE method is:

$${\hat{SI}}_{AE}=\underset{a}{\mathrm{argmin}}\;L(a+\frac{b}{t}+\frac{c}{t^{2}};\;SI_{naive}(t)),$$

where *L* is the loss (squared errors) for fitting *SI*_*naive*_*(t)* with the defined function. The analytical derivation of the bias, however, was based on an asymptotic expansion, which is applicable only to the asymptotic sampling regime [10], but not to small sample sizes. Moreover, fitting the data using this type of function results in divergence to infinity when *t* approaches zero, while the actual naïve information is bounded.

### Bounded asymptotic extrapolation (BAE)

Since the naïve information is bounded, we modified the function used for fitting the naïve MI as a function of sample size to the form:

$$SI_{naive}(t)=a+\frac{b}{1+ct}.$$

The parameter *a* is equal to the extrapolation of the function to infinity and serves as the estimated SI. Thus, the estimated SI based on the BAE method is:

$${\hat{SI}}_{BAE}=\underset{a}{\mathrm{argmin}}\;L(a+\frac{b}{1+ct};\;SI_{naive}(t))$$

To evaluate the validity of the BAE method, we applied similar principles using alternative bounded and monotonically decreasing function types, e.g., an exponential function, and compared their performance (S14 Fig).

### Validating the bias-correction methods using simulated data

To obtain ground-truth information values on which the accuracy of our bias-correction methods can be directly tested, we simulated data with similar coding properties to those observed in the hippocampal data. Specifically, we simulated neuronal activity for N = 100 place cells per session, with a lognormal distribution of peak firing rates, and a distribution of active time bins that matches the distribution in the real data. We then simulated neuronal responses with Gaussian tuning curves that evenly tile the stimulus space, with a tuning width distribution that matches the distribution in the data. To match the stimulus statistics, we used the behavioral data from our linear track experiments. At each time bin, neuronal responses were drawn from a Poisson distribution (in agreement with the assumption of the SI derivation), where the average rate of each neuron (in that bin) was determined by its tuning curve and the current position of the mouse. Then, we used the simulated neuronal responses to estimate the tuning curves, and the naïve calculation of SI and the bias-correction methods were applied to these estimated tuning curves, exactly as for the real data. Since the ground-truth tuning curve of each neuron is equivalent to the estimated tuning curves from an infinite amount of data, the true SI was obtained by applying the naïve calculation of SI to the ground-truth tuning curves. The true MI was calculated using the ground-truth joint and marginal probabilities to observe a certain number of spikes and for the mouse to be in a specific spatial bin, under the assumption of Poisson firing statistics. In addition, we simulated spike trains with variability in the firing rate distributions that does not follow Poisson firing statistics. We systematically changed the variance of the firing rate distributions without changing the average firing rates in response to a given value of the stimulus. We used the Binomial distribution to obtain firing rate distributions with sub-Poisson statistics (Fano factor < 1) and the negative Binomial distribution for supra-Poisson statistics (Fano factor > 1). For this analysis, the time bin was set to 200 msec and the average firing rates were 10 times higher compared to the real data, to allow us to obtain sub-Poisson variability without changing the average rate.

### Validating the bias-correction methods using experimental data

Since there is no ground truth for the information content of real data, there is no straightforward way to validate the accuracy of our bias-correction methods. Instead, we used the ability to track the same neurons across multiple days with Ca^2+^ imaging data [4], and concatenated six 20-minute-long sessions into a single 2-hour-long session. Only place cells that were active in at least five out of the six sessions were used for this validation. Assuming that for a sample size of 2 hours the bias is negligible (Fig 6A), we used the naïve SI of the concatenated session as an approximation of the ground truth. Then, we applied the different bias-correction methods to subsamples of 20 minutes from the concatenated session. Finally, we evaluated the accuracy of the bias-correction methods against the concatenated session naïve SI.

### Allen brain observatory data set

To apply our bias-correction methods to estimate non-spatial information content, we analyzed two-photon Ca^2+^ imaging data from the publicly available Allen Brain Observatory (https://observatory.brain-map.org/visualcoding) [42]. We analyzed neuronal activity in all of the mice in the data set that were imaged in the primary visual cortex (V1), from the excitatory Cre lines (Cux2-CreERT2, Emx1-IRES-Cre, Fezf2-CreER, Nr5a1-Cre, Ntsr1-Cre_GN220, Rbp4-Cre_KL100, Rorb-IRES2-Cre, Scnn1a-Tg3-Cre, Slc17a7-IRES2-Cre, Tlx3-Cre_PL56), during the repeated presentation of drifting gratings (presented in ‘session A’). The drifting-grating stimuli consisted of a combination of five different temporal frequencies and eight different movement directions (two directions for each of four different orientations). Each stimulus presentation persisted for two seconds, followed by one second of a gray screen between stimuli. For each cell, fluorescence change traces (ΔF_(t)_/F_0_) were extracted using automated, structural region of interest (ROI)-based methods (see Allen Institute white paper for details). We identified Ca^2+^ events by searching each trace for local maxima that had a peak amplitude higher than four times the entire trace absolute median, while including only the frames that showed an increase in Ca^2+^ transients relative to their previous frame (during the rise time). All the ΔF_(t)_/F_0_ values in the frames that passed the event-detection criteria were set to 1, and the rest were set to 0. We then calculated the number of active frames for each stimulus presentation and calculated the average firing rate as a function of the different stimuli. Finally, we calculated the joint information carried about the temporal frequency and direction of movement of the gratings for each cell and applied our bias-correction methods to the data. Overall, data were taken from 94 experiments, each corresponding to imaging from a single mouse.

### Statistical analysis

The changes in the average SI during learning were tested using repeated-measures ANOVA. Post hoc pairwise comparison tests were performed using the FDR method for correction of multiple comparisons. The relationship between the average SI and the within-field peak firing rate was tested using linear regression. Differences between groups of paired samples were tested using a matched-pairs t-test.

## Supporting information

S1 Fig Comparison between random permutations and cyclic permutations for the identification of significant place cells and the estimation of shuffle SI. (A) A higher percentage of cells significantly modulated by position (place cells) are obtained with random shuffles compared to cyclic shuffles (matched-pairs two-sided t-test_(8)_, t = 8.1, p = 4.0·10^−5^). (B-C) Percentage of place cells (mean ± SEM) as a function of the sample duration (B) or number of active time bins (C) for randomly shuffled (black), or cyclically shuffled (gray) data. (D) Naïve SI (mean ± SD) as a function of sample duration for real (blue), randomly shuffled (black), or cyclically shuffled (gray) data. (E-F) The naïve SI (mean ± SD) as a function of the average firing rates (E) or number of active time bins (F) for real (blue), randomly shuffled (black), or cyclically shuffled (gray) data. To compare the shuffle SI in the same cells between random and cyclic shuffles, place cells were identified using the cyclic permutation test in all cases in D-F. Data were averaged across N = 9 mice. ***p < 0.001.
(TIF)

S2 Fig Estimated SI shows a similar bias as a function of the sample duration or number of track traversals. (A) Naïve SI (mean ± SD) as a function of the sample duration or number of track traversals for real (blue/cyan) and shuffled (black/gray) data. (B) The entropy of the mouse position (mean ± SD) as a function of sample duration or track traversals. Data are shown up to the minimal number of track traversals completed across all mice. Since the number of track traversals differs across sessions and mice, a linear interpolation was performed on the SI/entropy as a function of sample duration to allow its visualization on the same x-axis as the number of track traversals. Data were averaged across N = 9 mice.
(TIF)

S3 Fig The upward bias in the naïve SI expressed in bit/sec. (A-C) Naïve SI (mean ± SEM) for real (blue) and shuffled (black) data, expressed in bit/sec, decreases as a function of the sampling duration (A) and increases with the average firing rate (B) or number of active time bins (C). (D-F) The ratio between the shuffle and the naïve SI (mean ± SEM) decreases with sample duration (D), average firing rate (E) or number of active time bins (F). The decrease in this ratio indicates a smaller relative contribution of the bias to the calculated SI for the more active cells. Data were averaged across N = 9 mice.
(TIF)

S4 Fig The upward bias in the naïve MI. (A-C) Naïve MI (mean ± SEM) for real (blue) and shuffled (black) data decreases as a function of the sampling duration (A) and increases with the average firing rate (B) or number of active time bins (C). (D-F) The ratio between the shuffle MI and the naïve MI mean ± SEM) decreases with sample duration (D), average firing rate (E) or number of active time bins (F). The decrease in this ratio indicates a smaller relative contribution of the bias to the calculated MI for the more active cells. Data were averaged across N = 9 mice.
(TIF)

S5 Fig Dispersion of shuffle SI (and SI Z-score with respect to the shuffles) decreases (increases) with sample size and activity levels. (A-C) Standard deviation of the shuffle SI across shuffling repetitions (mean ± SD) decreases as a function of the sampling duration (A) average firing rate (B) and number of active time bins (C). (D-F) The Z-score of the naïve SI with respect to the shuffle SI (mean ± SD) increases as a function of the sampling duration (D) average firing rate (E) and number of active time bins (F). Data were averaged across N = 9 mice.
(TIF)

S6 Fig The bias in the naïve SI decreases with the average firing rate and number of active time bins. (A-B) Average naïve SI (mean ± SD) of simulated place cells (blue), the true SI (cyan) and the bias (gray) as a function of the average firing rate (A) or number of active time bins (B). Data were averaged across N = 9 simulations. Each simulation corresponds to behavioral data from a different mouse and consists of 100 simulated place cells.
(TIF)

S7 Fig The ratio between the bias in the naïve SI and shuffle SI decreases with tuning specificity, but not with sample size. (A) The ratio between the bias in the naïve SI and shuffle SI versus the true SI of each simulated place cell, for small sample sizes (sample duration = 1 minute). Data pooled from N = 9 simulations, each corresponding to behavioral data from a different mouse. (B) The bias ratio (mean ± SEM) decreases as a function of the true SI for a sample size of 1 minute (blue), 5 minutes (cyan) and 20 minutes (green). (C) The bias ratio (mean ± SEM) is maintained between a sample size of 1 minute versus 5 minutes (cyan) and 20 minutes (green). y = x is shown in black. Data in B-C were averaged across N = 9 simulations. (D) The average bias ratio as a function of the sample duration, for each of the nine simulations (gray) and the average across simulations (black). (E) The ratio between the bias in the naïve SI and shuffle SI versus the true SI (expressed in bit/sec) of each simulated place cell, for small sample sizes (sample duration = 1 minute). Data were pooled from N = 9 simulations. (F) The average bias ratio (for SI expressed in bit/sec) as a function of the sample duration, for each of the nine simulations (gray) and the average across simulations (black). Note that the assumption of the sample size’s independent bias ratio is less accurate when SI is expressed in bit/sec compared to bit/spike, consistent with the dependence of the bias on the number of relevant response bins (response bins with non-zero probability), which is mostly related to tuning specificity, but not to the firing rates of the neurons. Each simulation corresponds to behavioral data from a different mouse and consists of 100 simulated place cells.
(TIF)

S8 Fig The bias in the naïve SI and the accuracy of the bias-correction methods as a function of tuning specificity. (A) Naïve SI (mean ± SD) of simulated place cells (blue), the bias (gray), and the average true SI (cyan), as a function of the true SI. Inset, zoom-in on the bias. (B) Absolute estimation bias (mean ± SEM) as a function of the cells’ true SI, for SR (red), SSR (magenta), AE (orange), and BAE (green). Data were averaged across N = 9 simulations. Each simulation corresponds to behavioral data from a different mouse and consists of 100 simulated place cells.
(TIF)

S9 Fig The accuracy of the SSR method is maintained across a wide range of *t*_*1*_ subsample durations. (A-B) Demonstration of the SSR method using a subsample duration *t*_*1*_ = 60 sec (A) and a subsample duration *t*_*1*_ = 600 sec (B). Estimated SI as a function of sample duration for the naïve calculation (blue), shuffle (black), the SSR estimation (magenta), and the true SI (cyan). Data in A-B show the mean across 100 cells from one example simulation. (C) Absolute estimation bias (mean ± SEM) as a function of subsample duration *t*_*1*_ for SR (red) and SSR (magenta). The calculation of SR relies only on the full sample duration and therefore it does not depend on subsample duration *t*_*1*_ as the SSR method. Data were averaged across N = 9 simulations. Each simulation corresponds to behavioral data from a different mouse and consists of 100 simulated place cells.
(TIF)

S10 Fig Bias-correction methods applied to the naïve calculation of SI expressed in bit/sec. (A) Applying shuffle-based bias-correction methods to the naïve SI (blue) of simulated place cells expressed in bit/sec. SSR (magenta) is more accurate than SR (red) in estimating the true SI (cyan). Shuffle SI is shown in black. (B) Applying extrapolation-based bias-correction methods to the naïve SI (blue) of simulated place cells expressed in bit/sec. BAE (green) is more accurate than AE (orange) in estimating the true SI (cyan). Data in A-B show the mean across 100 cells from one example simulation. (C-D) The estimated SI (C) and the absolute estimation bias (D) as a function of the sample duration (mean ± SEM) for the naïve calculation (blue), SR (red), SSR (magenta), AE (orange), and BAE (green). SSR and BAE yield smaller biases than SR and AE when estimating the true SI. Inset, zoom-in on the estimated SI in C shows that the SSR method shifts from underestimating to slightly overestimating the SI at specific sample durations, resulting in the non-monotonous absolute estimation bias as a function of sample duration found in D. Data in C-D were averages across N = 9 simulations. Each simulation corresponds to behavioral data from a different mouse and consists of 100 simulated place cells.
(TIF)

S11 Fig Bias-correction methods applied to the naïve MI. (A) Applying shuffle-based bias-correction methods to the naïve MI (blue) of simulated place cells. SSR (magenta) is more accurate than SR (red) in estimating the true MI (cyan). Shuffle MI is shown in black. (B) Applying extrapolation-based bias-correction methods to the MI of simulated place cells. BAE (green) is more accurate than AE (orange) in estimating the true MI (cyan). Data in A-B show the mean across 100 cells from one example simulation. (C-D) The estimated MI (C) and the absolute estimation bias (D) as a function of the sample duration (mean ± SEM) for the naïve calculation (blue), SR (red), SSR (magenta), AE (orange), and BAE (green) methods. SSR and BAE yield smaller biases compared to SR and AE when estimating the true MI. Data in C-D show the averages across N = 9 simulations. Each simulation corresponds to behavioral data from a different mouse and consists of 100 simulated place cells.
(TIF)

S12 Fig The rate the bias decreases with sample duration is higher for cells with higher activity levels. (A-B) Bias decay rate (approximated by the parameter *c* in the fitting function *a+b/(1+ct)* of the BAE method) is higher for cells with higher firing rates (A) or number of active time bins (B). This result is consistent with the smaller bias observed for the more active cells (Fig 2C and 2D). Parameter *c* can be considered a good approximation of the rate in which the bias decays for large sample durations *t*, allowing one to capture each cell’s rate with a single value that is independent of sample size. Furthermore, since for larger values of *c*, the function of the form *a+b/(1+ct)* is closer to the form of *a+b/t*, this result may explain why the difference between the performance of the AE and BAE methods is smaller for the more active cells. Data were averaged across N = 9 simulations. Each simulation corresponds to behavioral data from a different mouse and consists of 100 simulated place cells.
(TIF)

S13 Fig BAE yields a more accurate estimation than AE even when only large subsamples are used. (A-C) AE and BAE estimation as a function of the minimal subsample duration *t*_*min*_ when applied to simulated data. (A-B) AE estimation (orange) and BAE estimation (green), based on 5–100% (A) or 50–100% (B) of the full data size. Data in A-B show the mean across 100 cells from one example simulation. (C) Absolute estimation bias (mean ± SEM) as a function of the minimal subsample duration *t*_*min*_ for AE (orange) and BAE (green). While using only larger subsamples improved the performance of the AE method, the BAE method was more accurate in estimating the true SI across all values of *t*_*min*_. Data were averaged across N = 9 simulations. Each simulation corresponds to behavioral data from a different mouse and consists of 100 simulated place cells. (D-F) AE and BAE estimation as a function of the minimal subsample duration *t*_*min*_ when applied to real data. (D-E) AE estimation (orange) and BAE estimation (green), based on 5–100% (D) or 50–100% (E) of the full data size. Data in D-E show the mean across cells from one example mouse. (F) Normalized fit residuals (mean ± SEM) as a function of the minimal subsample duration *t*_*min*_ for AE (orange) and BAE (green). Inset, zoom-in on the normalized fit residuals for the larger values of *t*_*min*_. While using only larger subsamples improved the fit accuracy of the AE method, the BAE method yielded a more accurate fit across all values of *t*_*min*_. Data were averaged across N = 9 mice.
(TIF)

S14 Fig Alternative bounded and monotonically decreasing functions do not accurately fit the bias in the naïve SI. (A-D) Fitting the naïve SI (blue) of simulated data using different types of bounded and monotonically decreasing functions with three free parameters. BAE (green; C) yields a more accurate estimation of the true SI (cyan) compared to an exponential function (red; A) or a power law (magenta; B). Data in A-C show the mean across 100 cells from one example simulation. (D) Absolute estimation bias (mean ± SEM) across the different bounded fitting functions. The BAE method is more accurate than exponential extrapolation (matched-pairs two-sided t-test_(8)_, t = 48.4, p = 3.7·10^−11^) and the power law extrapolation (matched-pairs two-sided t-test_(8)_, t = 8.3, p = 3.4·10^−5^). Data were averaged across N = 9 simulations. Each simulation corresponds to behavioral data from a different mouse and consists of 100 simulated place cells. (E-H) Fitting the naïve SI (blue) of real data using different types of bounded and monotonically decreasing functions with three free parameters. BAE (green; G) exhibits higher fit accuracy compared to an exponential function (red; E) or a power law (magenta; F). Normalized residuals for each fit are indicated. (H) Normalized residuals (mean ± SEM) in fitting the naïve SI as a function of sample duration across the different bounded fitting functions. The BAE method more accurately fits the data than an exponential fit (matched-pairs two-sided t-test_(8)_, t = 7.4, p = 7.4·10^−5^) and a power law fit (matched-pairs two-sided t-test_(8)_, t = 5.8, p = 4.1·10^−4^). Data were averaged across N = 9 mice. ***p < 0.001.
(TIF)

S15 Fig Correcting the bias in the naïve estimation of information content for firing rate distributions that do not follow Poisson statistics. (A) The true MI rate (solid cyan curve) and the true SI rate (dashed cyan curve) expressed in bit/sec as a function of the time bin, for 1,000 simulated neurons with Poisson firing statistics and firing rates that match the real data. For time bins that do not approach zero, the MI rate is lower than the SI rate due to redundancy between multiple spikes that occur within the same bin. (B) The true MI rate (solid cyan curve) and the true SI rate (dashed cyan curve) as a function of the Fano factor, for 1,000 simulated neurons with supra-Poisson variability (Fano factor > 1) in the distribution of firing rates. For Fano factors > 1, the SI rate overestimates the MI rate. The time bin was set to 50 msec, as in the real data. (C) Illustration of spike count distributions with a fixed average rate for sub-Poisson variability (Fano factor < 1; green), Poisson statistics (Fano factor = 1; blue) and supra Poisson variability (Fano factor > 1; red). A binomial distribution was used to obtain Fano factors < 1 and a negative binomial distribution was used to obtain Fano factors >1. Longer time bins or higher average firing rates compared to those observed in the data were required to obtain sub-Poisson variability without changing the average rate. (D) The average naïve MI (blue), true MI (cyan), bias (gray) and shuffle MI (black) as a function of the Fano factor. Inset, relative bias as a function of the Fano factor. The bias increases with the Fano factor. Note that the shuffle MI is considerably greater than zero even for small Fano factors, for which the bias is negligible. (E-F) The estimated MI (E) and the absolute estimation bias (F) as a function of the Fano factor for the naïve calculation (blue), SR (red), SSR (magenta), AE (orange), and BAE (green). Note that the SR method yields a large downward bias even for very low Fano factors, an outcome of the non-zero shuffle MI found in D. Data in D-F show the mean ± SEM across 1,000 simulated neurons. In D-F, the time bin was set to 200 msec, and the average firing rates were 10 times higher compared to the real data.
(TIF)

S16 Fig Subsampling the data recapitulates the results obtained from the bias-correction methods. (A) The estimated SI (mean ± SEM) as a function of the within-field peak firing rates for the naïve calculation (blue), SSR (magenta), BAE (green), and for the naïve calculation applied to subsampled data (dashed blue curve). SI increased with peak rate based on the naïve estimation applied to subsampled data (linear regression_(10)_, R^2^ = 0.97, slope = 0.05 ± 0.01, p = 1.8·10^−7^). The data from each cell were subsampled to obtain a fixed average firing rate across all cells. (B) The estimated SI (mean ± SEM) as a function of time in the experiment for the naïve calculation (blue), SSR (magenta), BAE (green), and for the naïve calculation applied to subsampled data (dashed blue curve). SI increased with learning for the naïve estimation applied to subsampled data (repeated-measures ANOVA_(7)_, F = 11.82, p = 3.9·10^−9^). The data from each session were subsampled to obtain a fixed number of track traversals across all sessions. (C) Naïve SI (mean) as a function of the sample duration during different learning sessions (colors). Data are shown up to the minimal number of track traversals completed across all sessions. For the analyses presented in panels B-C, only place cells with ≥ 10 active time bins in a given session were used. Data in A-C were averaged across N = 9 mice. Data in B-C for each familiarity level (day in the experiment) were averaged across the two environments. ***p < 0.001.
(TIF)
