# Supplementary figures and images for: Bias-free estimation of information content in temporally sparse neuronal activity
Source: PLoS Comput Biol. 2022 Feb 11;18(2):e1009832. doi: 10.1371/journal.pcbi.1009832 (PMC8836373; doi:10.1371/journal.pcbi.1009832)

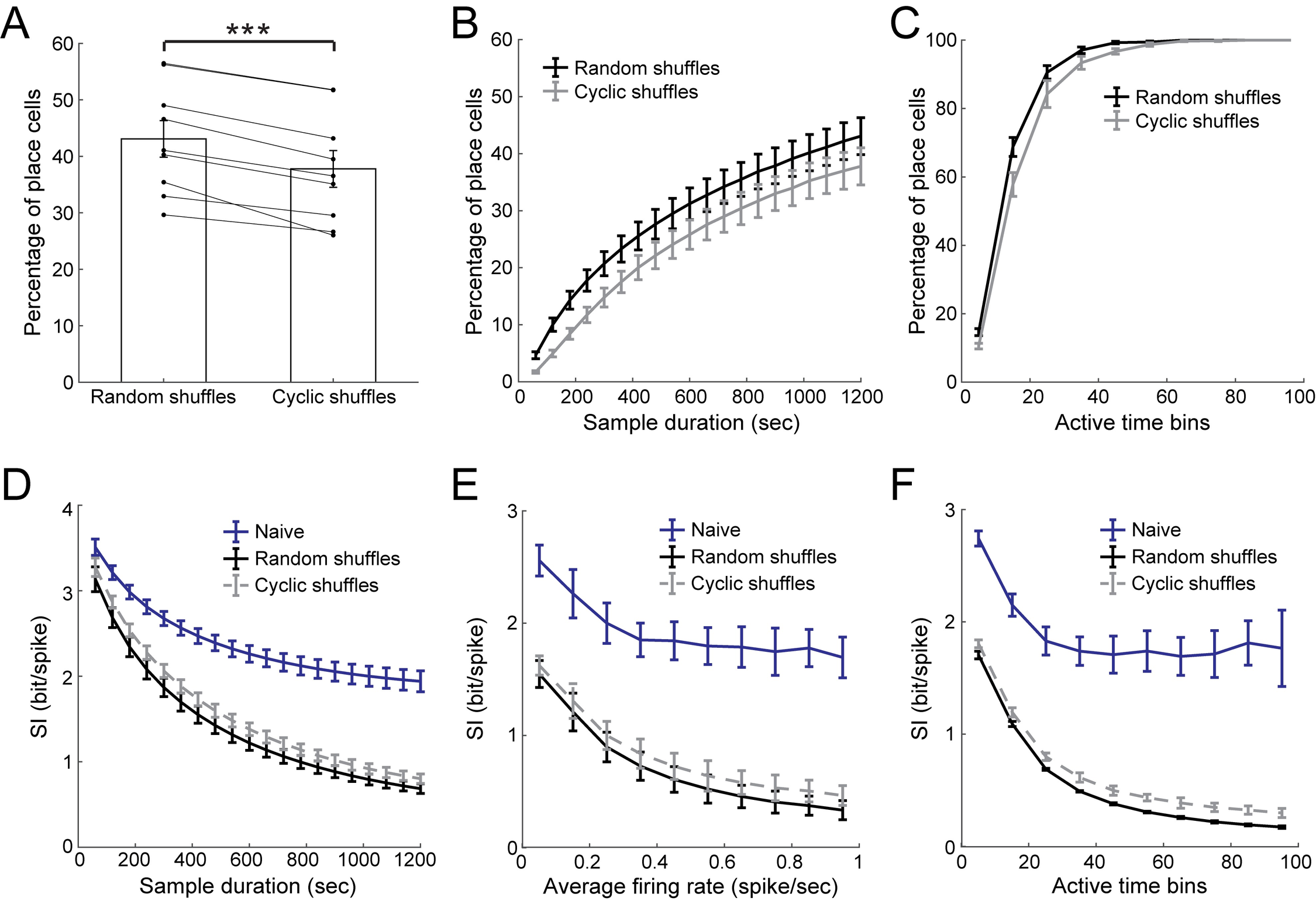

Supplement: S1 Fig — (A) A higher percentage of cells significantly modulated by position (place cells) are obtained with random shuffles compared to cyclic shuffles (matched-pairs two-sided t-test(8), t = 8.1, p = 4.0·10−5). (B-C) Percentage of place cells (mean ± SEM) as a function of the sample duration (B) or number of active time bins (C) for randomly shuffled (black), or cyclically shuffled (gray) data. (D) Naïve SI (mean ± SD) as a function of sample duration for real (blue), randomly shuffled (black), or cyclically shuffled (gray) data. (E-F) The naïve SI (mean ± SD) as a function of the average firing rates (E) or number of active time bins (F) for real (blue), randomly shuffled (black), or cyclically shuffled (gray) data. To compare the shuffle SI in the same cells between random and cyclic shuffles, place cells were identified using the cyclic permutation test in all cases in D-F. Data were averaged across N = 9 mice. ***p < 0.001. (TIF) [file pcbi.1009832.s001.tif]

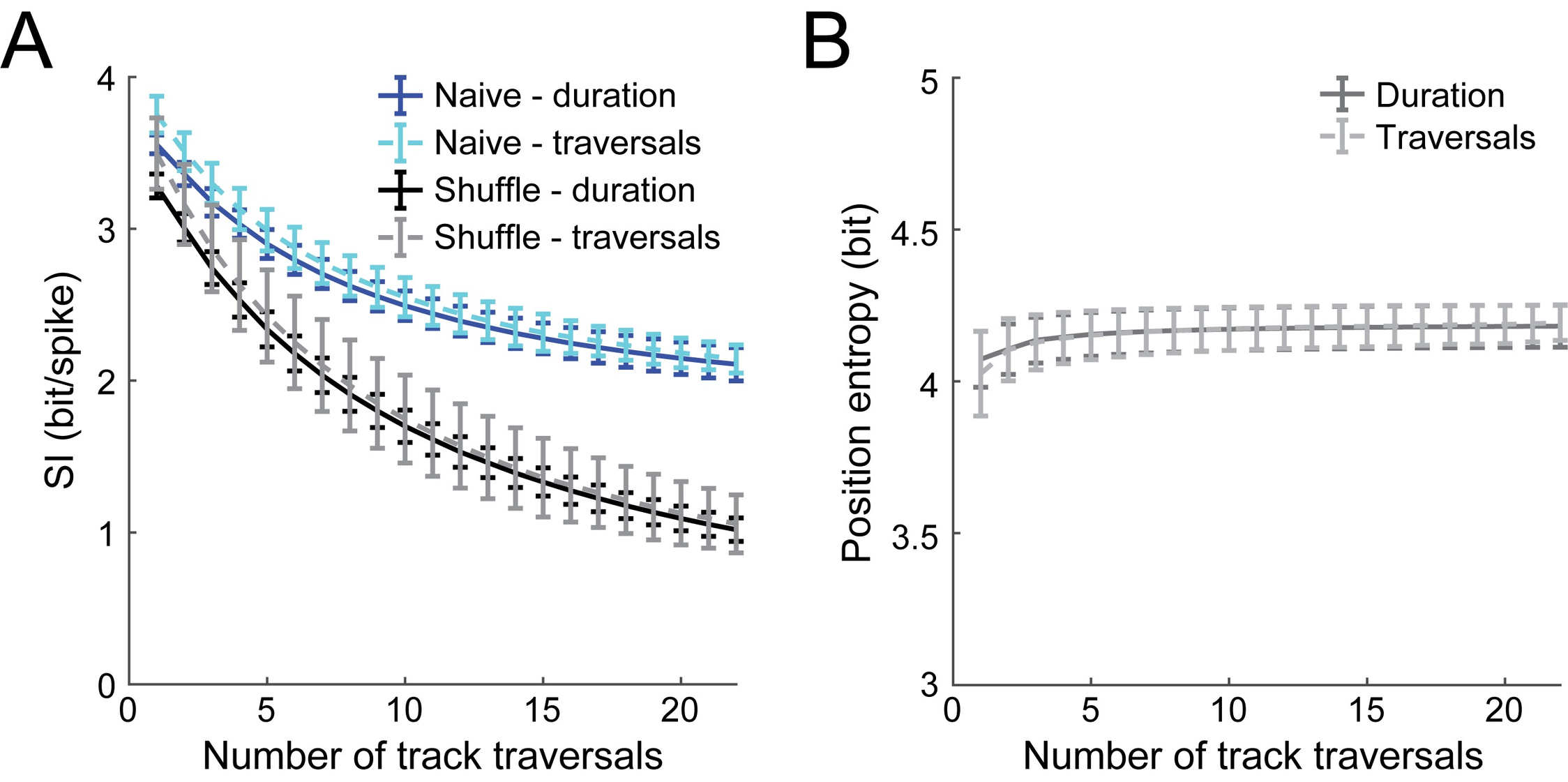

Supplement: S2 Fig — (A) Naïve SI (mean ± SD) as a function of the sample duration or number of track traversals for real (blue/cyan) and shuffled (black/gray) data. (B) The entropy of the mouse position (mean ± SD) as a function of sample duration or track traversals. Data are shown up to the minimal number of track traversals completed across all mice. Since the number of track traversals differs across sessions and mice, a linear interpolation was performed on the SI/entropy as a function of sample duration to allow its visualization on the same x-axis as the number of track traversals. Data were averaged across N = 9 mice. (TIF) [file pcbi.1009832.s002.tif]

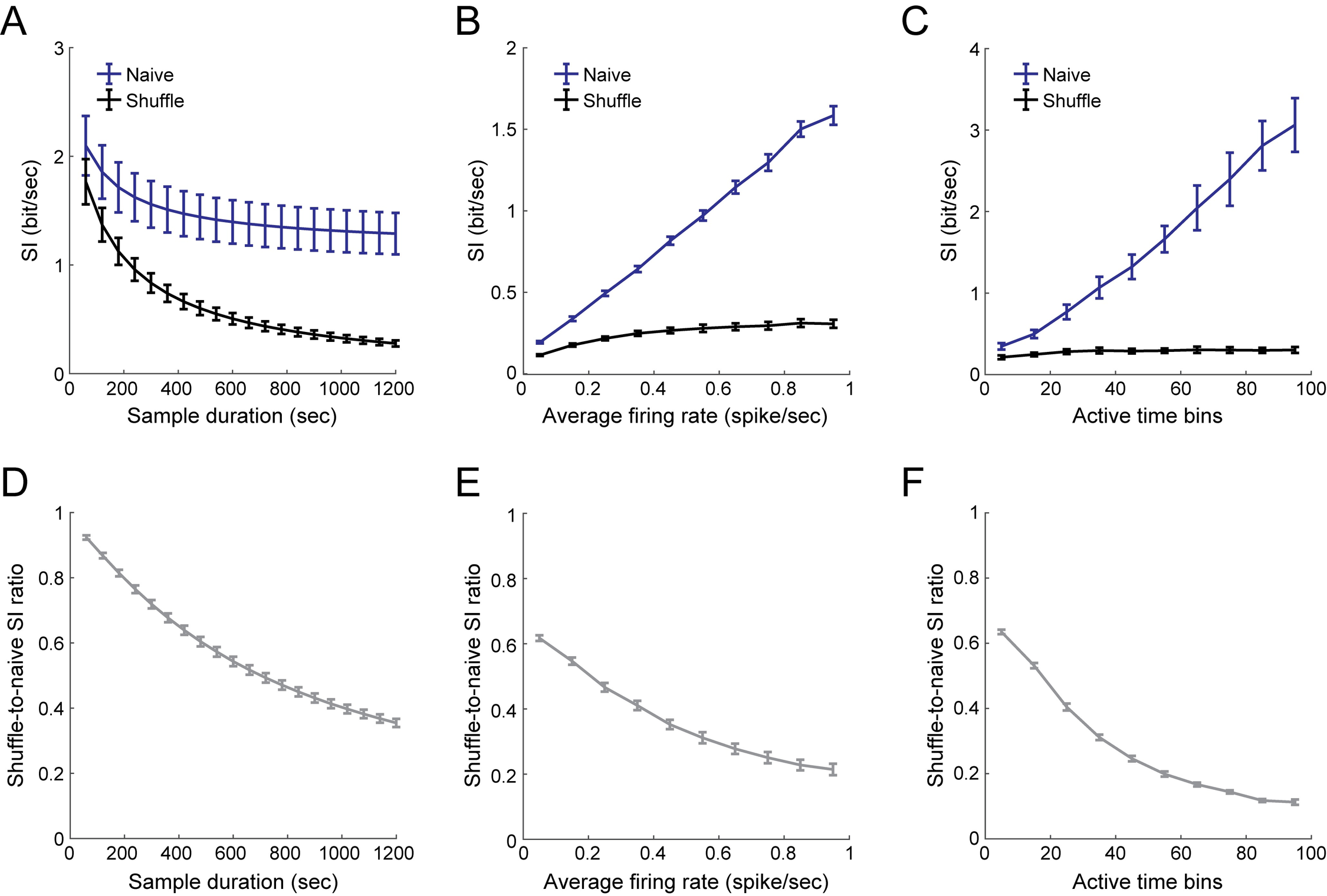

Supplement: S3 Fig — (A-C) Naïve SI (mean ± SEM) for real (blue) and shuffled (black) data, expressed in bit/sec, decreases as a function of the sampling duration (A) and increases with the average firing rate (B) or number of active time bins (C). (D-F) The ratio between the shuffle and the naïve SI (mean ± SEM) decreases with sample duration (D), average firing rate (E) or number of active time bins (F). The decrease in this ratio indicates a smaller relative contribution of the bias to the calculated SI for the more active cells. Data were averaged across N = 9 mice. (TIF) [file pcbi.1009832.s003.tif]

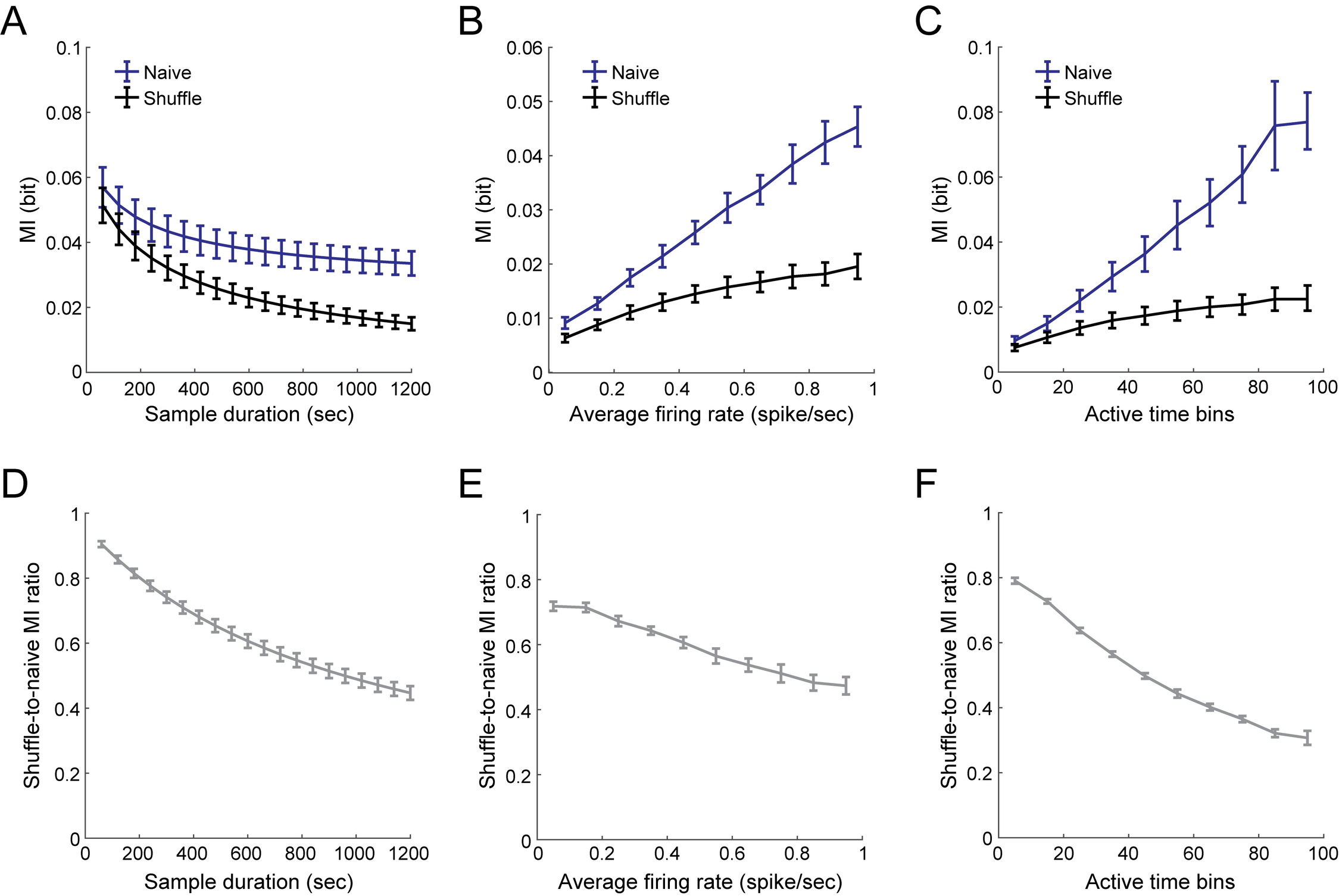

Supplement: S4 Fig — (A-C) Naïve MI (mean ± SEM) for real (blue) and shuffled (black) data decreases as a function of the sampling duration (A) and increases with the average firing rate (B) or number of active time bins (C). (D-F) The ratio between the shuffle MI and the naïve MI mean ± SEM) decreases with sample duration (D), average firing rate (E) or number of active time bins (F). The decrease in this ratio indicates a smaller relative contribution of the bias to the calculated MI for the more active cells. Data were averaged across N = 9 mice. (TIF) [file pcbi.1009832.s004.tif]

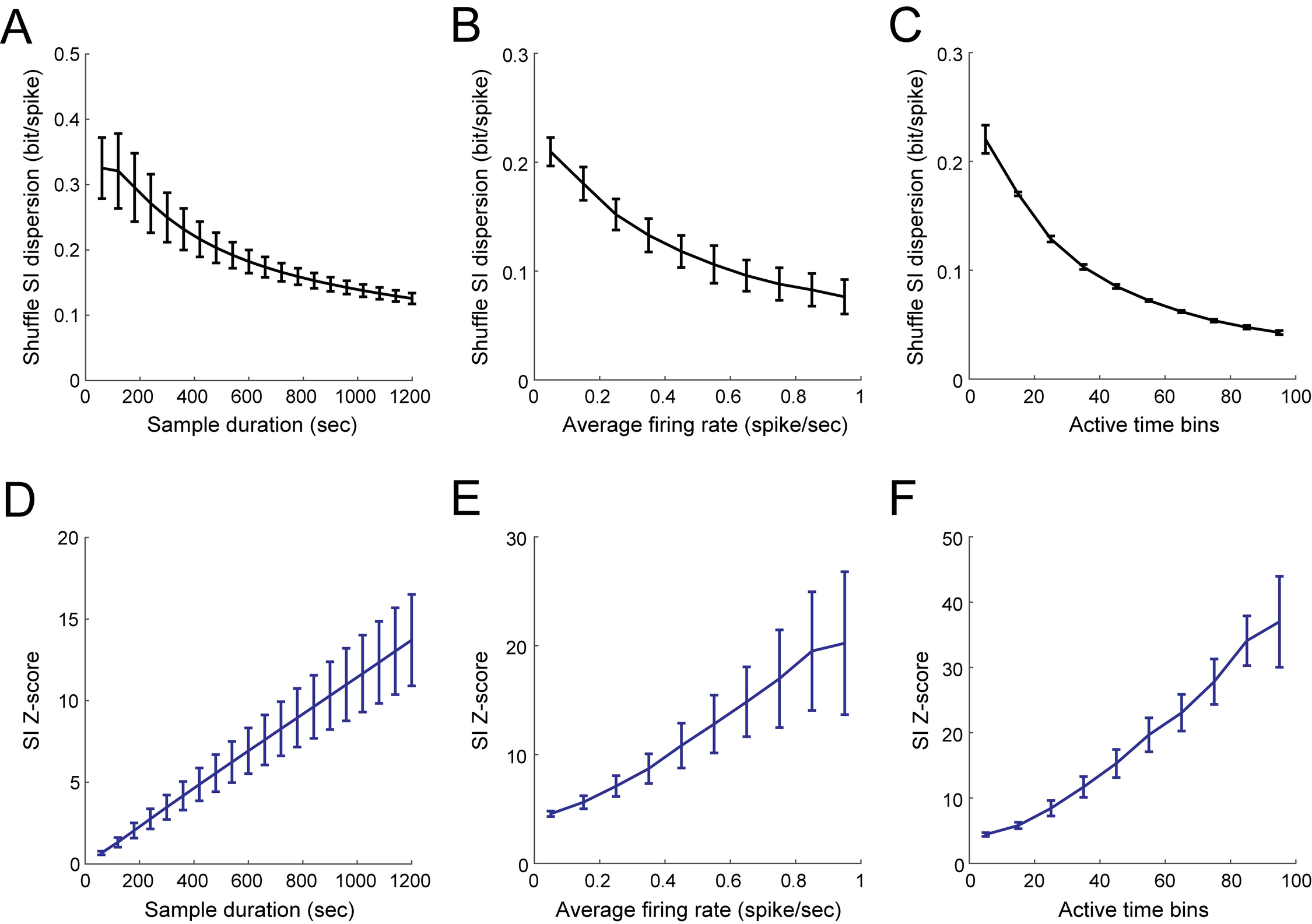

Supplement: S5 Fig — (A-C) Standard deviation of the shuffle SI across shuffling repetitions (mean ± SD) decreases as a function of the sampling duration (A) average firing rate (B) and number of active time bins (C). (D-F) The Z-score of the naïve SI with respect to the shuffle SI (mean ± SD) increases as a function of the sampling duration (D) average firing rate (E) and number of active time bins (F). Data were averaged across N = 9 mice. (TIF) [file pcbi.1009832.s005.tif]

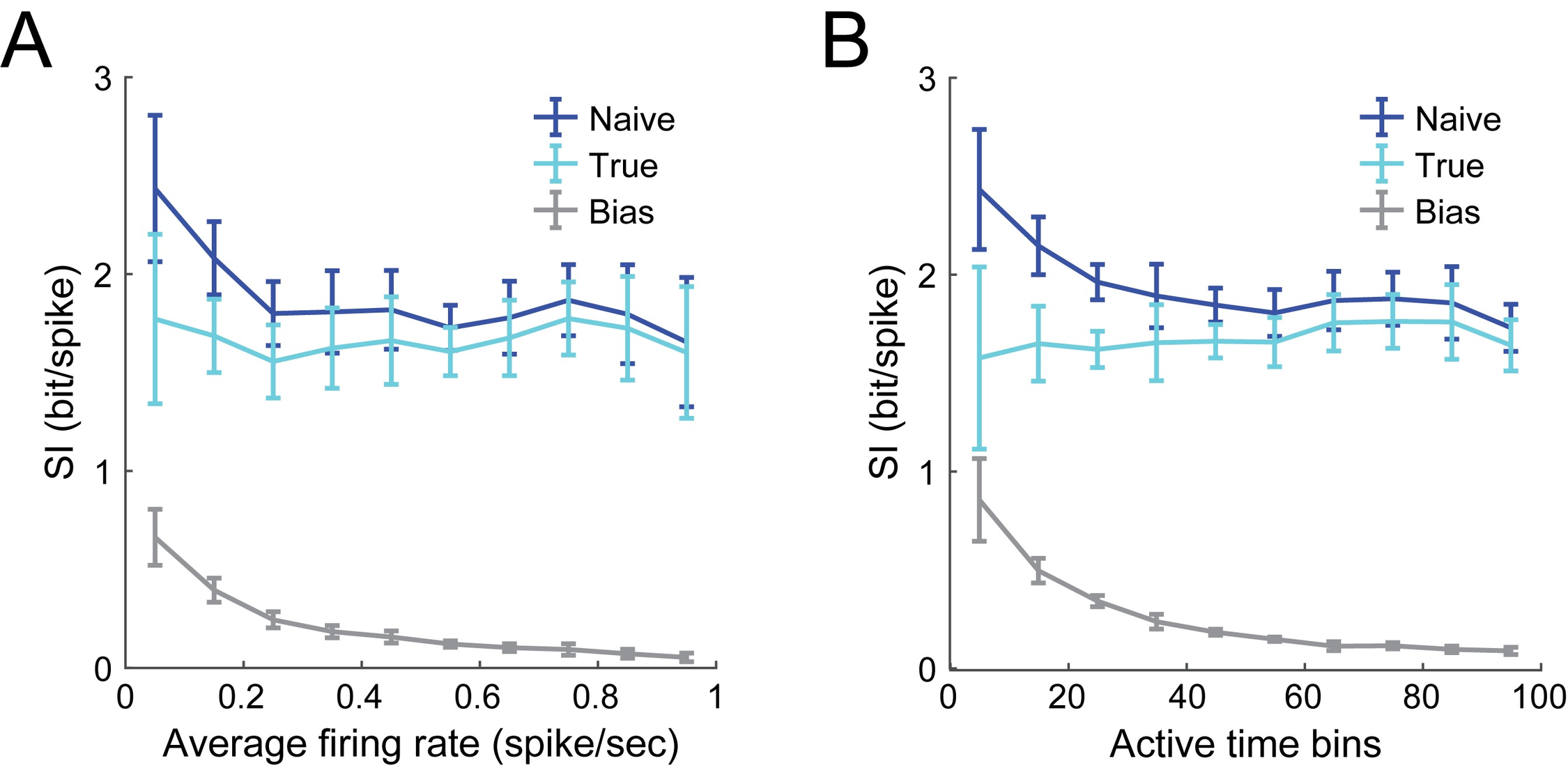

Supplement: S6 Fig — (A-B) Average naïve SI (mean ± SD) of simulated place cells (blue), the true SI (cyan) and the bias (gray) as a function of the average firing rate (A) or number of active time bins (B). Data were averaged across N = 9 simulations. Each simulation corresponds to behavioral data from a different mouse and consists of 100 simulated place cells. (TIF) [file pcbi.1009832.s006.tif]

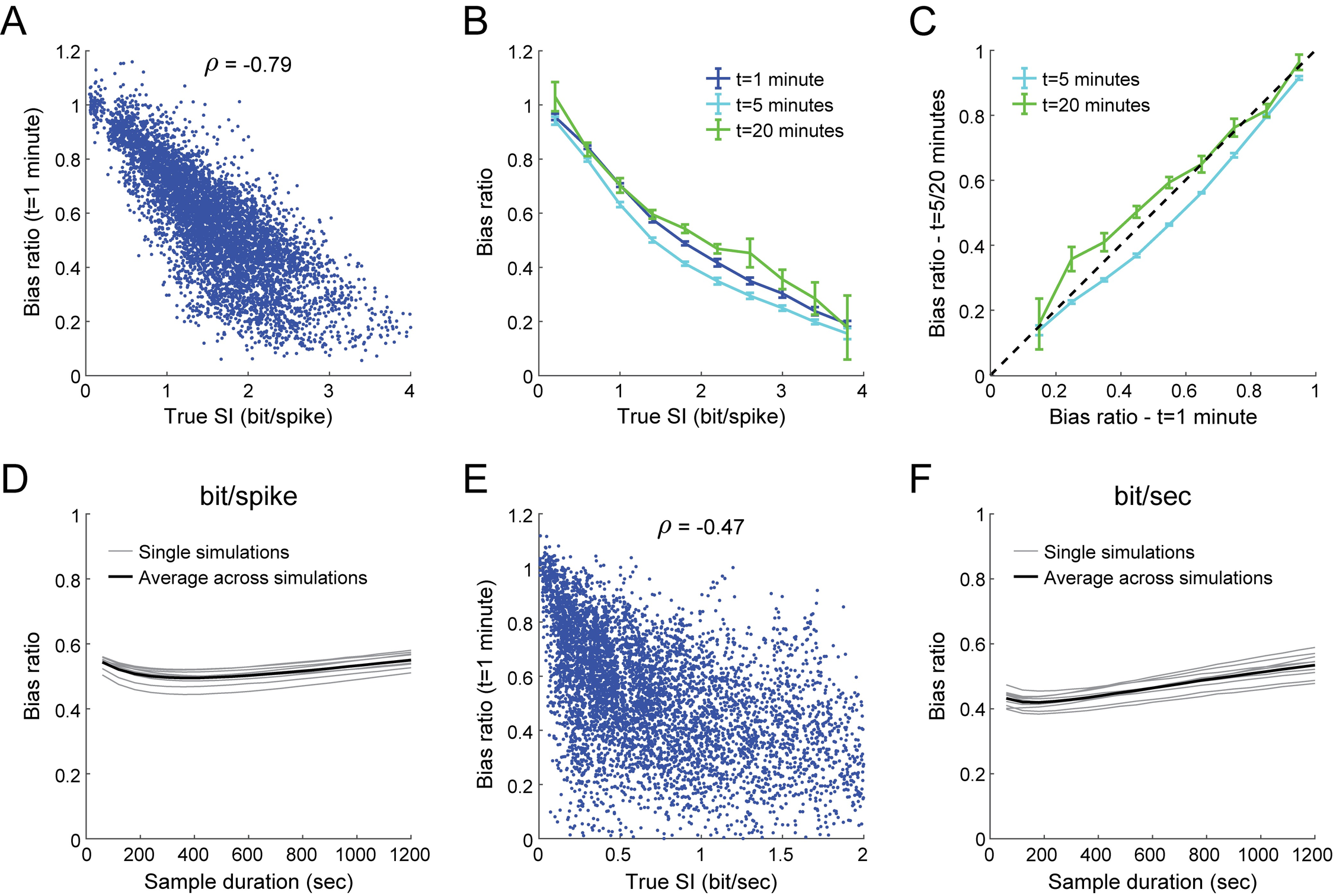

Supplement: S7 Fig — (A) The ratio between the bias in the naïve SI and shuffle SI versus the true SI of each simulated place cell, for small sample sizes (sample duration = 1 minute). Data pooled from N = 9 simulations, each corresponding to behavioral data from a different mouse. (B) The bias ratio (mean ± SEM) decreases as a function of the true SI for a sample size of 1 minute (blue), 5 minutes (cyan) and 20 minutes (green). (C) The bias ratio (mean ± SEM) is maintained between a sample size of 1 minute versus 5 minutes (cyan) and 20 minutes (green). y = x is shown in black. Data in B-C were averaged across N = 9 simulations. (D) The average bias ratio as a function of the sample duration, for each of the nine simulations (gray) and the average across simulations (black). (E) The ratio between the bias in the naïve SI and shuffle SI versus the true SI (expressed in bit/sec) of each simulated place cell, for small sample sizes (sample duration = 1 minute). Data were pooled from N = 9 simulations. (F) The average bias ratio (for SI expressed in bit/sec) as a function of the sample duration, for each of the nine simulations (gray) and the average across simulations (black). Note that the assumption of the sample size’s independent bias ratio is less accurate when SI is expressed in bit/sec compared to bit/spike, consistent with the dependence of the bias on the number of relevant response bins (response bins with non-zero probability), which is mostly related to tuning specificity, but not to the firing rates of the neurons. Each simulation corresponds to behavioral data from a different mouse and consists of 100 simulated place cells. (TIF) [file pcbi.1009832.s007.tif]

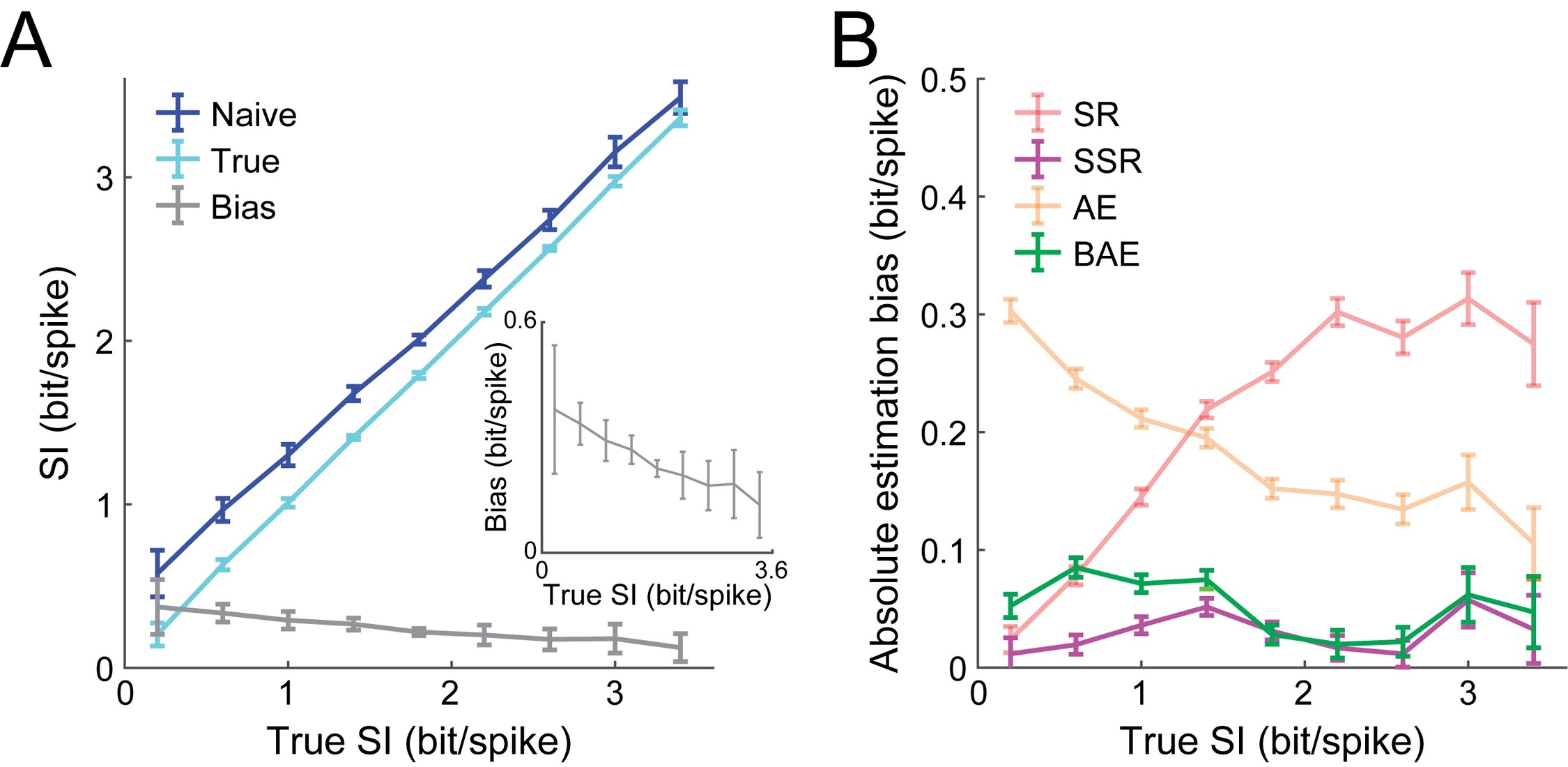

Supplement: S8 Fig — (A) Naïve SI (mean ± SD) of simulated place cells (blue), the bias (gray), and the average true SI (cyan), as a function of the true SI. Inset, zoom-in on the bias. (B) Absolute estimation bias (mean ± SEM) as a function of the cells’ true SI, for SR (red), SSR (magenta), AE (orange), and BAE (green). Data were averaged across N = 9 simulations. Each simulation corresponds to behavioral data from a different mouse and consists of 100 simulated place cells. (TIF) [file pcbi.1009832.s008.tif]

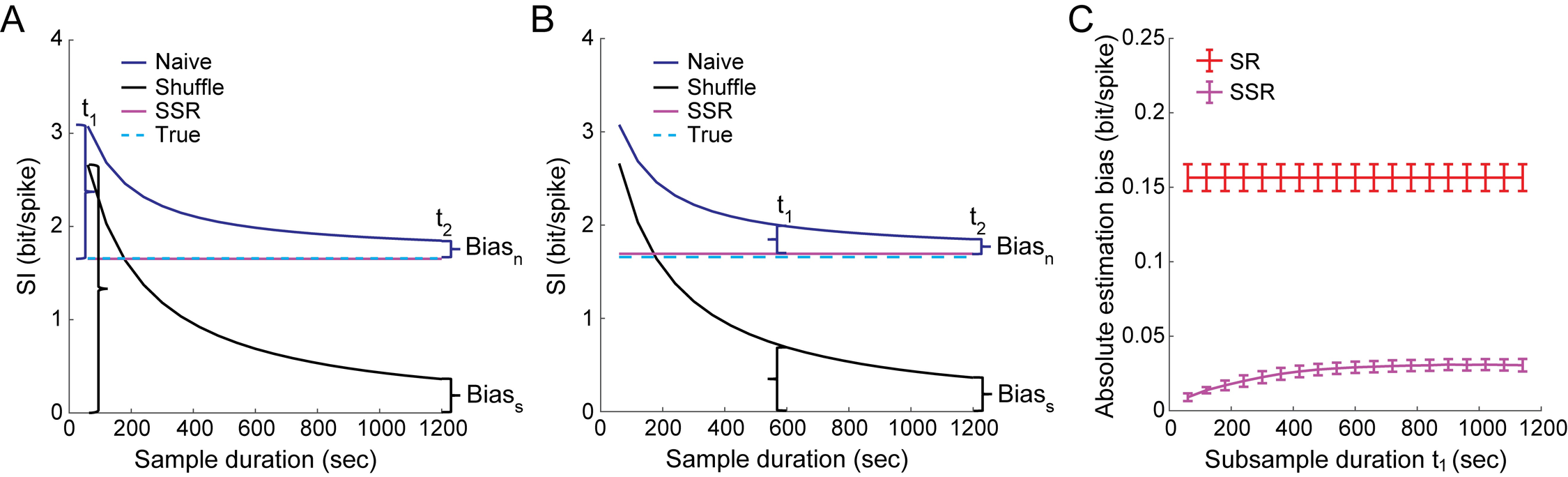

Supplement: S9 Fig — (A-B) Demonstration of the SSR method using a subsample duration t1 = 60 sec (A) and a subsample duration t1 = 600 sec (B). Estimated SI as a function of sample duration for the naïve calculation (blue), shuffle (black), the SSR estimation (magenta), and the true SI (cyan). Data in A-B show the mean across 100 cells from one example simulation. (C) Absolute estimation bias (mean ± SEM) as a function of subsample duration t1 for SR (red) and SSR (magenta). The calculation of SR relies only on the full sample duration and therefore it does not depend on subsample duration t1 as the SSR method. Data were averaged across N = 9 simulations. Each simulation corresponds to behavioral data from a different mouse and consists of 100 simulated place cells. (TIF) [file pcbi.1009832.s009.tif]

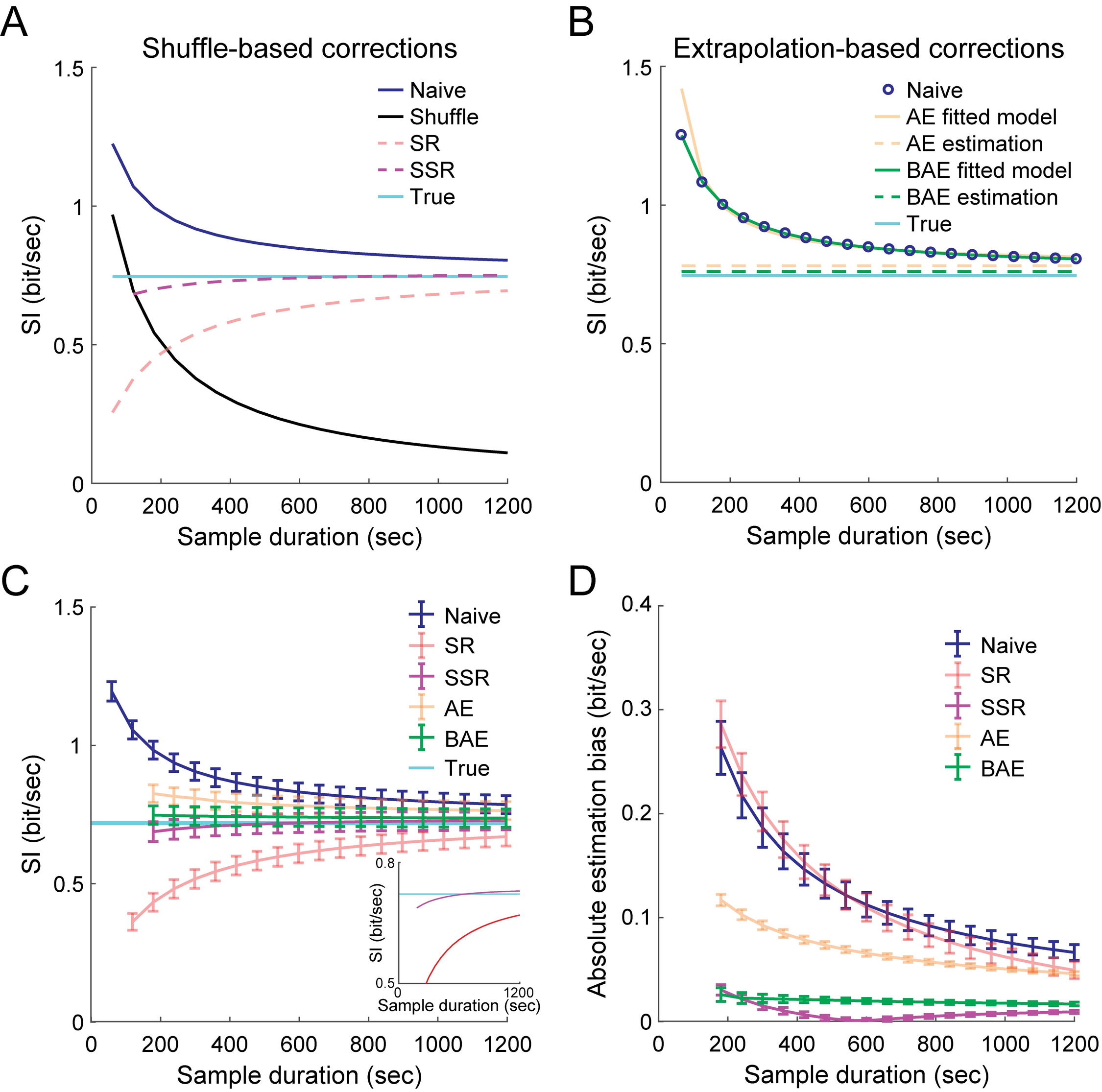

Supplement: S10 Fig — (A) Applying shuffle-based bias-correction methods to the naïve SI (blue) of simulated place cells expressed in bit/sec. SSR (magenta) is more accurate than SR (red) in estimating the true SI (cyan). Shuffle SI is shown in black. (B) Applying extrapolation-based bias-correction methods to the naïve SI (blue) of simulated place cells expressed in bit/sec. BAE (green) is more accurate than AE (orange) in estimating the true SI (cyan). Data in A-B show the mean across 100 cells from one example simulation. (C-D) The estimated SI (C) and the absolute estimation bias (D) as a function of the sample duration (mean ± SEM) for the naïve calculation (blue), SR (red), SSR (magenta), AE (orange), and BAE (green). SSR and BAE yield smaller biases than SR and AE when estimating the true SI. Inset, zoom-in on the estimated SI in C shows that the SSR method shifts from underestimating to slightly overestimating the SI at specific sample durations, resulting in the non-monotonous absolute estimation bias as a function of sample duration found in D. Data in C-D were averages across N = 9 simulations. Each simulation corresponds to behavioral data from a different mouse and consists of 100 simulated place cells. (TIF) [file pcbi.1009832.s010.tif]

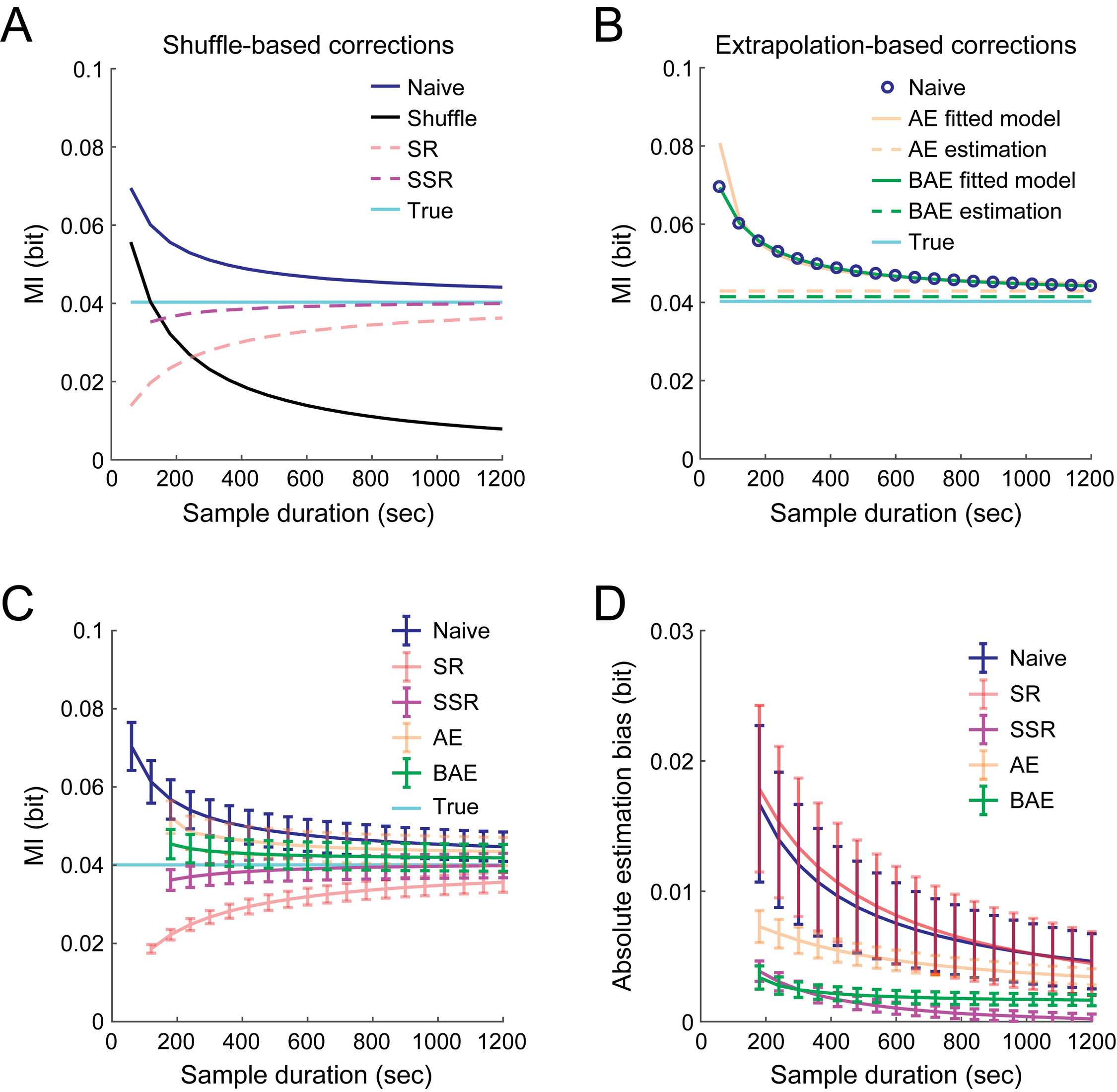

Supplement: S11 Fig — (A) Applying shuffle-based bias-correction methods to the naïve MI (blue) of simulated place cells. SSR (magenta) is more accurate than SR (red) in estimating the true MI (cyan). Shuffle MI is shown in black. (B) Applying extrapolation-based bias-correction methods to the MI of simulated place cells. BAE (green) is more accurate than AE (orange) in estimating the true MI (cyan). Data in A-B show the mean across 100 cells from one example simulation. (C-D) The estimated MI (C) and the absolute estimation bias (D) as a function of the sample duration (mean ± SEM) for the naïve calculation (blue), SR (red), SSR (magenta), AE (orange), and BAE (green) methods. SSR and BAE yield smaller biases compared to SR and AE when estimating the true MI. Data in C-D show the averages across N = 9 simulations. Each simulation corresponds to behavioral data from a different mouse and consists of 100 simulated place cells. (TIF) [file pcbi.1009832.s011.tif]

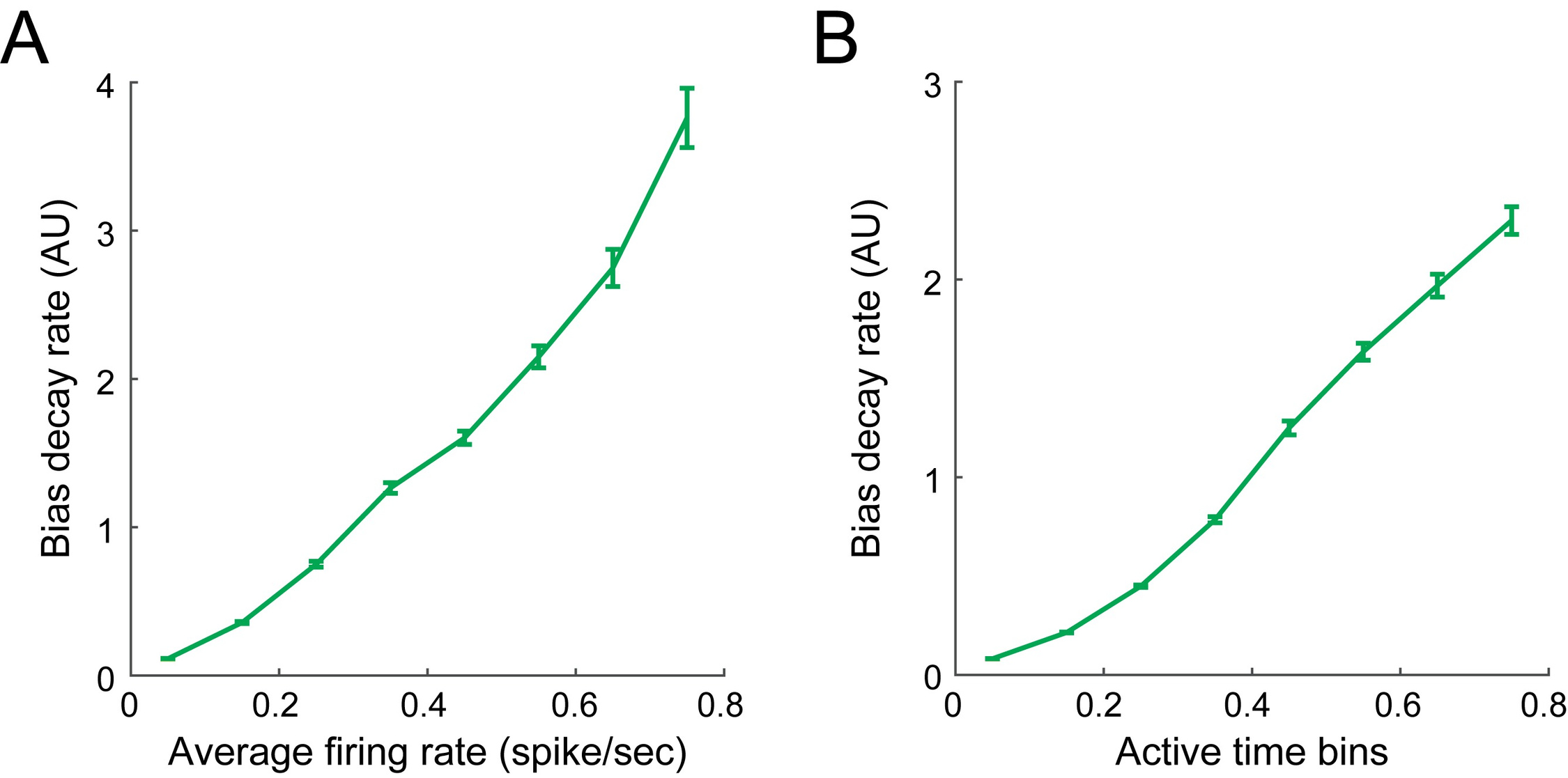

Supplement: S12 Fig — (A-B) Bias decay rate (approximated by the parameter c in the fitting function a+b/(1+ct) of the BAE method) is higher for cells with higher firing rates (A) or number of active time bins (B). This result is consistent with the smaller bias observed for the more active cells (Fig 2C and 2D). Parameter c can be considered a good approximation of the rate in which the bias decays for large sample durations t, allowing one to capture each cell’s rate with a single value that is independent of sample size. Furthermore, since for larger values of c, the function of the form a+b/(1+ct) is closer to the form of a+b/t, this result may explain why the difference between the performance of the AE and BAE methods is smaller for the more active cells. Data were averaged across N = 9 simulations. Each simulation corresponds to behavioral data from a different mouse and consists of 100 simulated place cells. (TIF) [file pcbi.1009832.s012.tif]

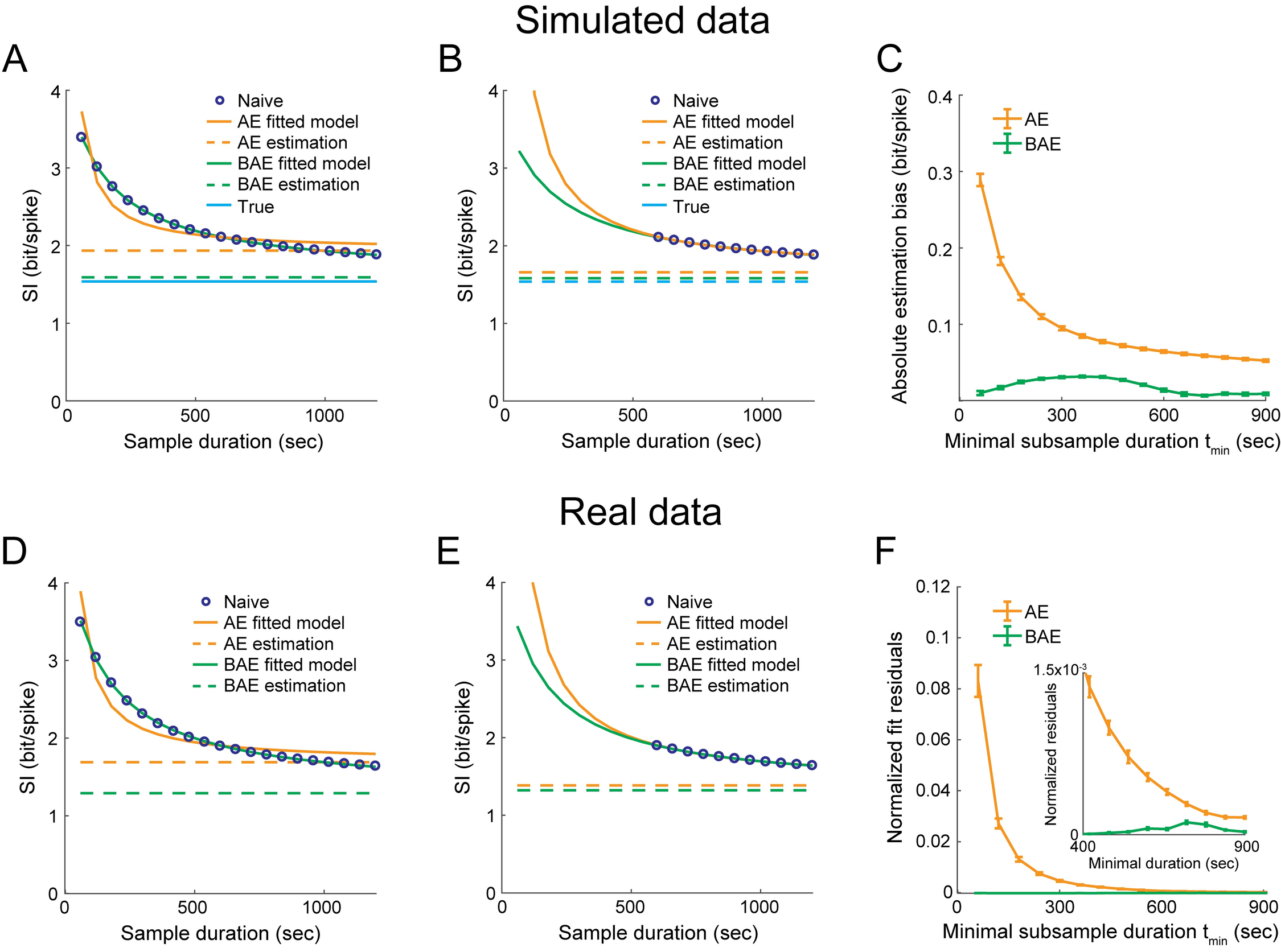

Supplement: S13 Fig — (A-C) AE and BAE estimation as a function of the minimal subsample duration tmin when applied to simulated data. (A-B) AE estimation (orange) and BAE estimation (green), based on 5–100% (A) or 50–100% (B) of the full data size. Data in A-B show the mean across 100 cells from one example simulation. (C) Absolute estimation bias (mean ± SEM) as a function of the minimal subsample duration tmin for AE (orange) and BAE (green). While using only larger subsamples improved the performance of the AE method, the BAE method was more accurate in estimating the true SI across all values of tmin. Data were averaged across N = 9 simulations. Each simulation corresponds to behavioral data from a different mouse and consists of 100 simulated place cells. (D-F) AE and BAE estimation as a function of the minimal subsample duration tmin when applied to real data. (D-E) AE estimation (orange) and BAE estimation (green), based on 5–100% (D) or 50–100% (E) of the full data size. Data in D-E show the mean across cells from one example mouse. (F) Normalized fit residuals (mean ± SEM) as a function of the minimal subsample duration tmin for AE (orange) and BAE (green). Inset, zoom-in on the normalized fit residuals for the larger values of tmin. While using only larger subsamples improved the fit accuracy of the AE method, the BAE method yielded a more accurate fit across all values of tmin. Data were averaged across N = 9 mice. (TIF) [file pcbi.1009832.s013.tif]

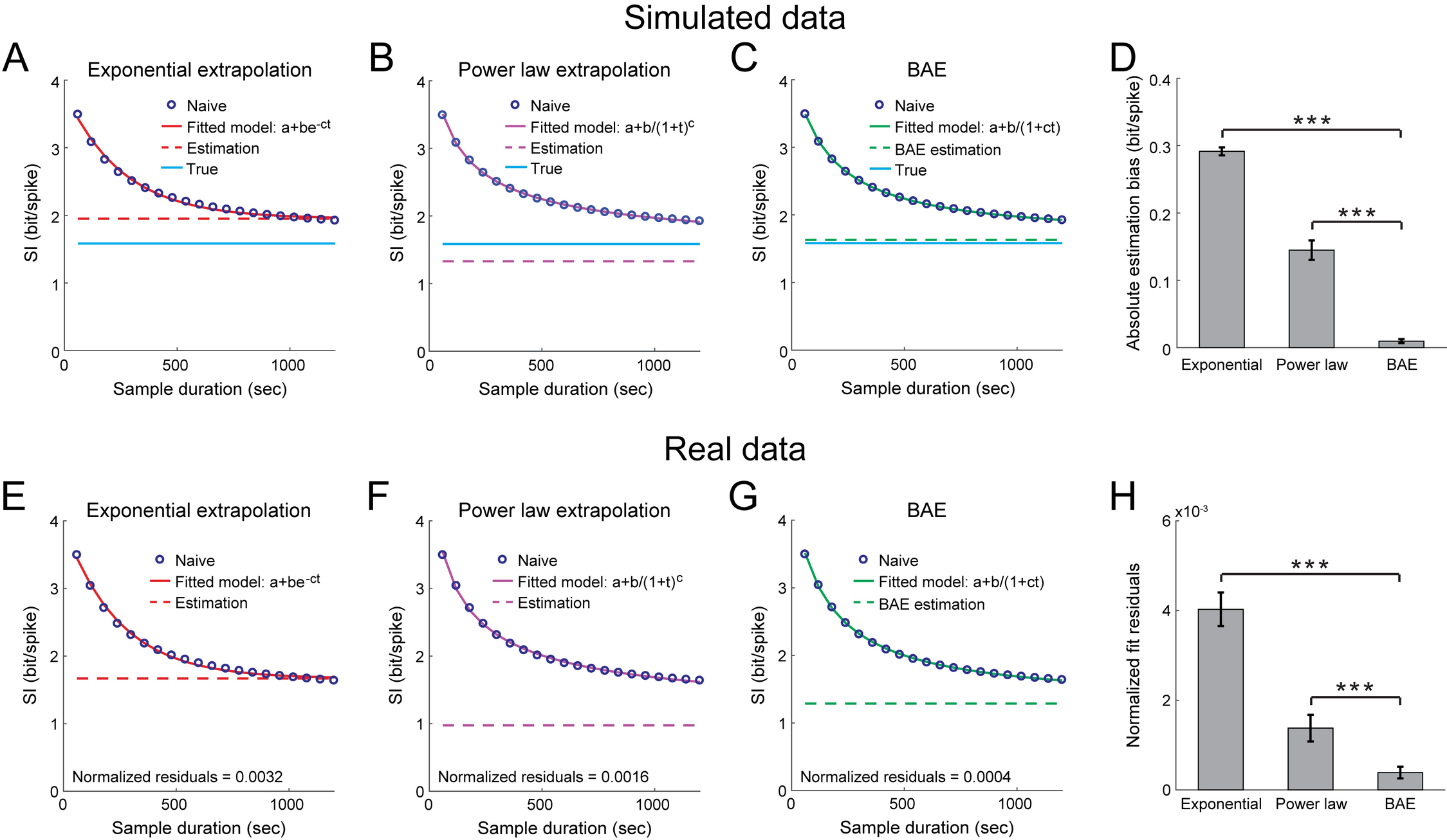

Supplement: S14 Fig — (A-D) Fitting the naïve SI (blue) of simulated data using different types of bounded and monotonically decreasing functions with three free parameters. BAE (green; C) yields a more accurate estimation of the true SI (cyan) compared to an exponential function (red; A) or a power law (magenta; B). Data in A-C show the mean across 100 cells from one example simulation. (D) Absolute estimation bias (mean ± SEM) across the different bounded fitting functions. The BAE method is more accurate than exponential extrapolation (matched-pairs two-sided t-test(8), t = 48.4, p = 3.7·10−11) and the power law extrapolation (matched-pairs two-sided t-test(8), t = 8.3, p = 3.4·10−5). Data were averaged across N = 9 simulations. Each simulation corresponds to behavioral data from a different mouse and consists of 100 simulated place cells. (E-H) Fitting the naïve SI (blue) of real data using different types of bounded and monotonically decreasing functions with three free parameters. BAE (green; G) exhibits higher fit accuracy compared to an exponential function (red; E) or a power law (magenta; F). Normalized residuals for each fit are indicated. (H) Normalized residuals (mean ± SEM) in fitting the naïve SI as a function of sample duration across the different bounded fitting functions. The BAE method more accurately fits the data than an exponential fit (matched-pairs two-sided t-test(8), t = 7.4, p = 7.4·10−5) and a power law fit (matched-pairs two-sided t-test(8), t = 5.8, p = 4.1·10−4). Data were averaged across N = 9 mice. ***p < 0.001. (TIF) [file pcbi.1009832.s014.tif]

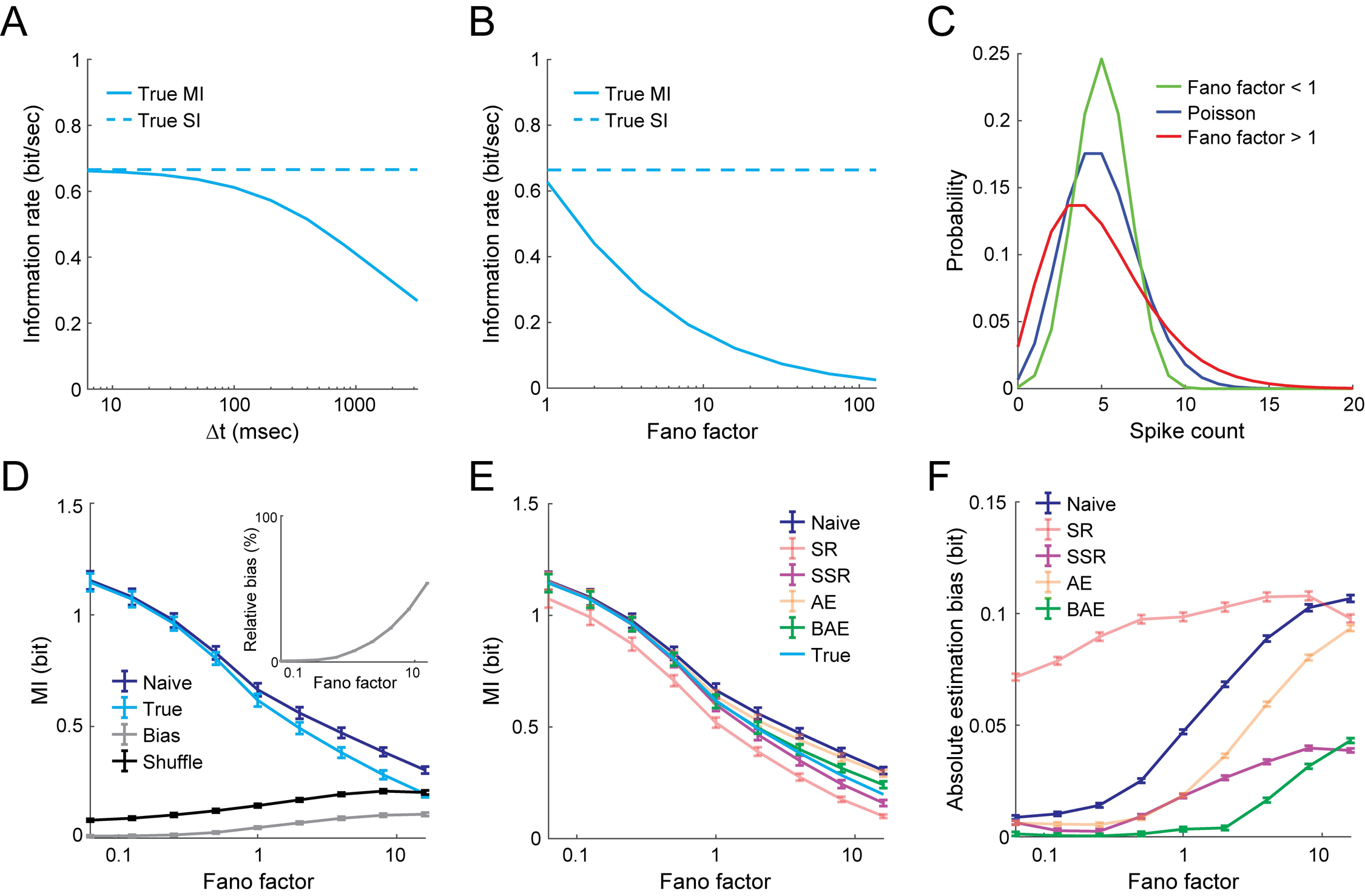

Supplement: S15 Fig — (A) The true MI rate (solid cyan curve) and the true SI rate (dashed cyan curve) expressed in bit/sec as a function of the time bin, for 1,000 simulated neurons with Poisson firing statistics and firing rates that match the real data. For time bins that do not approach zero, the MI rate is lower than the SI rate due to redundancy between multiple spikes that occur within the same bin. (B) The true MI rate (solid cyan curve) and the true SI rate (dashed cyan curve) as a function of the Fano factor, for 1,000 simulated neurons with supra-Poisson variability (Fano factor > 1) in the distribution of firing rates. For Fano factors > 1, the SI rate overestimates the MI rate. The time bin was set to 50 msec, as in the real data. (C) Illustration of spike count distributions with a fixed average rate for sub-Poisson variability (Fano factor < 1; green), Poisson statistics (Fano factor = 1; blue) and supra Poisson variability (Fano factor > 1; red). A binomial distribution was used to obtain Fano factors < 1 and a negative binomial distribution was used to obtain Fano factors >1. Longer time bins or higher average firing rates compared to those observed in the data were required to obtain sub-Poisson variability without changing the average rate. (D) The average naïve MI (blue), true MI (cyan), bias (gray) and shuffle MI (black) as a function of the Fano factor. Inset, relative bias as a function of the Fano factor. The bias increases with the Fano factor. Note that the shuffle MI is considerably greater than zero even for small Fano factors, for which the bias is negligible. (E-F) The estimated MI (E) and the absolute estimation bias (F) as a function of the Fano factor for the naïve calculation (blue), SR (red), SSR (magenta), AE (orange), and BAE (green). Note that the SR method yields a large downward bias even for very low Fano factors, an outcome of the non-zero shuffle MI found in D. Data in D-F show the mean ± SEM across 1,000 simulated neurons. In D-F, the time bin [file pcbi.1009832.s015.tif]

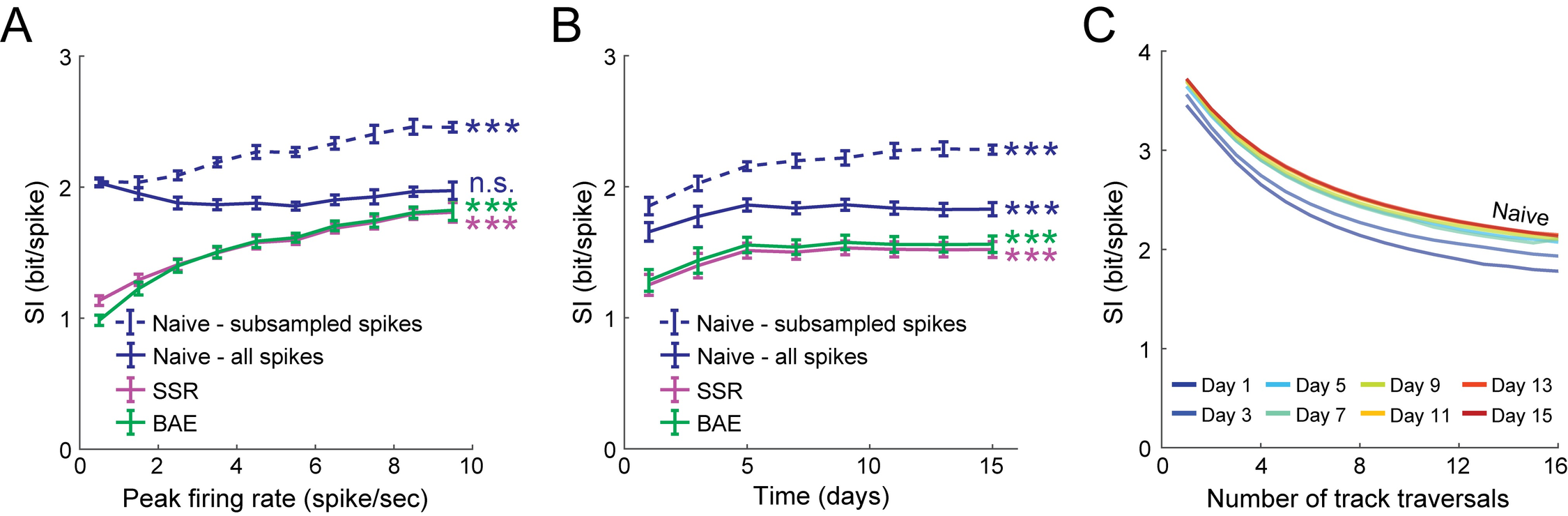

Supplement: S16 Fig — (A) The estimated SI (mean ± SEM) as a function of the within-field peak firing rates for the naïve calculation (blue), SSR (magenta), BAE (green), and for the naïve calculation applied to subsampled data (dashed blue curve). SI increased with peak rate based on the naïve estimation applied to subsampled data (linear regression(10), R2 = 0.97, slope = 0.05 ± 0.01, p = 1.8·10−7). The data from each cell were subsampled to obtain a fixed average firing rate across all cells. (B) The estimated SI (mean ± SEM) as a function of time in the experiment for the naïve calculation (blue), SSR (magenta), BAE (green), and for the naïve calculation applied to subsampled data (dashed blue curve). SI increased with learning for the naïve estimation applied to subsampled data (repeated-measures ANOVA(7), F = 11.82, p = 3.9·10−9). The data from each session were subsampled to obtain a fixed number of track traversals across all sessions. (C) Naïve SI (mean) as a function of the sample duration during different learning sessions (colors). Data are shown up to the minimal number of track traversals completed across all sessions. For the analyses presented in panels B-C, only place cells with ≥ 10 active time bins in a given session were used. Data in A-C were averaged across N = 9 mice. Data in B-C for each familiarity level (day in the experiment) were averaged across the two environments. ***p < 0.001. (TIF) [file pcbi.1009832.s016.tif]
